# International climate adaptation assistance: Assessing public support in Switzerland

**DOI:** 10.1371/journal.pone.0317344

**Published:** 2025-02-12

**Authors:** Liliana Andonova, Agnese Zucca, Simon Montfort, Nives Dolšak, Aseem Prakash

**Affiliations:** 1 Graduate Institute of International and Development Studies Geneva, Geneva, Switzerland; 2 École Polytechnique Fédérale de Lausanne, Lausanne, Switzerland; 3 University of Washington, Seattle, Washington, United States of America; Hankuk University of Foreign Studies, REPUBLIC OF KOREA

## Abstract

Lower-income countries account for a small share of accumulated greenhouse gas emissions but are highly vulnerable to climate-induced events. In response, industrialized higher-income countries, the major contributors to greenhouse gas stock, have pledged policy packages to support developing countries to adapt to climate change. Foreign aid and international migration often figure prominently in such packages. We employ a survey-embedded conjoint experiment to assess public support in Switzerland for international climate assistance packages which consist of six attributes: (1) the country receiving the package (Algeria, Kenya, Bangladesh, and the Philippines); (2) the volume of Swiss bilateral climate aid to this country; (3) the number of climate migrants from this country in Switzerland; (4) types of extreme weather event this country faces; (5) Swiss trade with this country; and (6) the country’s record of voting with Switzerland in the United Nations Security Council. We find that while Swiss respondents are indifferent to aid volume, their support for the policy package diminishes as the number of migrants increases. Respondents support policy packages for countries that trade with and vote alongside Switzerland in the Security Council. Respondents also have country-specific preferences: they support assistance to the Philippines, disfavor Algeria, and are indifferent to Kenya and Bangladesh. Ideology, cultural beliefs, and benchmarking with peer countries of Global North or past Swiss aid and immigration records do not change support for the policy package.

## Introduction

Global climate policy has an important domestic component. As Putnam’s [1] two-level game framework suggests, leaders take positions on global issues while being watchful of domestic reactions. The global-local connection has been debated in the context of climate mitigation policy. Historically the lack of progress on climate policy was largely attributed to global free riding. Increasingly, scholars recognize the important role of domestic distributional conflicts in impeding decarbonization [2–5], and alternatively, the significance of domestic political incentives for stimulating transnational climate initiatives [6,7].

In this paper, we extend the domestic-global connection to the study of climate adaptation politics. A well-established literature examines public support for adaptation at the domestic and local levels [8–11]. We contribute to this literature by examining domestic support for global climate adaptation assistance [12–14]. More specifically, we examine to what extent and in which ways domestic support for overseas climate adaptation among donor countries might depend on how citizens view the relationship of their own country with the country that will receive adaptation assistance. Thus, our paper provides a new direction to the study of climate politics by suggesting that while climate mitigation might be impeded by distributional implications, climate adaptation might be influenced by domestic perceptions of countries that receive adaptation support.

Historically, climate policy research focused predominantly on mitigation [15]. This was probably motivated by the concern that a focus on adaptation might reduce incentives to invest in mitigation; a moral hazard problem [16]. Some noted that adaptation was a “taboo” in the climate discourse [17]. While adaptation has slowly begun to feature more in climate discussions, it is not clear how it might be financed. The United Nations Framework Convention on Climate Change (UNFCCC) recognizes that advanced industrialized countries bear substantial responsibility for climate mitigation due to their significant contribution to the accumulated stock of greenhouse gases (GHG). Importantly, “shared but differentiated responsibility” should hold for adaptation as well. Because climate change is already in motion, Global North countries have a responsibility to support adaptation in Global South countries, which bear a disproportionate burden of climate change due to their high levels of exposure to climate risks along with meager resources to address them [18,19]. Thus, scholars note that climate adaptation has an important justice component [20]. The reality is that while Global North countries have made pledges at international forums to support climate adaptation overseas, the actual appropriations have fallen short [21]. Moreover, it is not clear what counts as adaptation aid, and whether Global North countries are double counting development aid as adaptation aid [22].

Why are Global North countries showing inadequate commitment to overseas climate aid? In part, this might be due to its low policy salience or low public support among their citizens. Moreover, most governments, even in Global North countries, struggle with managing multiple priorities within a limited budget. While opinion polls suggest that a majority of the population in the Global North is worried about climate change and wants governments to take action it is less clear if they are willing to support spending that creates adaptation benefits overseas.

This paper seeks to advance the understanding of factors driving domestic support for overseas adaptation aid [13,14]. While the foreign (development) aid literature explores public support in donor countries [23] and the implications for different ways of disbursing aid [24], the subject of public support for climate adaptation aid remains relatively underexplored (exceptions include Gampfer et al. [25] and Uji et al. [26]). More specifically, we examine how international issue linkages and domestic politics might interplay to shape public support for bilateral climate adaptation aid.

We build on Uji et al. [26], by asking survey respondents to choose between policy packages that include different levels of two forms of adaptation assistance: adaptation finance and accepting climate migrants. While Uji et al. examined public support in Japan using a conjoint experiment, we modified their instrument to the Swiss context. This allows us to make several contributions. First, the replication of the methodology in another donor country expands the comparative understanding of the factors that shape public support for overseas adaptation assistance in the form of adaptation aid and acceptance of climate migrants, as well as the overall external validity of the results. Second, we replicate the methodology in a political context where immigration is a highly salient issue, namely Switzerland and Europe more broadly [27–29], while migration is not a highly salient issue in Japan [26]. Moreover, aid and migration politics seem to be closely linked in Europe. Unlike Japan, European countries have sought to manage migrant flows by providing sizeable aid to some countries (especially on Africa’s Mediterranean coast) [30]. This means that the financing aspect of climate adaptation aid and immigration is likely to be more salient in the European context, especially with the vocal opposition from right-wing parties to climate mitigation and immigration. The implication is that the political context in which the two forms of adaptation (migration and aid) are discussed differs in Japan and Switzerland, and their comparative understanding is of paramount significance for the future of adaptation aid at large. Finally, from a theoretical perspective, the study of public support for adaptation aid in Switzerland provides an opportunity to elaborate theoretically on the two-level linkages between international collaboration on strategic issues and domestic support for adaptation aid.

The rest of the paper proceeds as follows. The theoretical section elaborates on the logic of adaptation policy packages and outlines a set of hypotheses about public support. Section 3 provides a more detailed justification of the case selection and specifies the methodology of data collection and analysis. Section 4 presents the results and discussion before we conclude with implications for policy and future research.

## Theoretical framework

International climate adaptation assistance is embedded in the broader debate on foreign aid and immigration. Because individuals seldom evaluate issues in isolation, we adopt a policy package approach in theorizing and evaluating public support for climate adaptation assistance to specific countries. This implies that public support for bilateral adaptation aid could be influenced by factors such as immigration from this country, Swiss trade with this country (instrumental motivations), how this country votes with Switzerland in the UN Security Council (convergence of policy positions in international forums), the location of the country (geographical bias), and the type of extreme weather event this country faces (empathy with countries with similar problems, or likely media salience of specific events).

To identify different dimensions of our policy package we elaborate on the idea that issue linkages could influence public support for bilateral assistance. Building on Uji et al. [26] we ask respondents to consider packages that allow them to take into account climate migration and levels of adaptation financing as two forms of adaptation assistance. The logic is that exposure of Global South countries to climate-related extreme weather events and their economic, infrastructure, and displacement impacts can be viewed as a “push factor” [31], which encourages individuals and households to emigrate overseas. Adaptation assistance can work via two mechanisms. Financial aid could enhance the resilience of the local community to extreme weather events (think of drought-proofing agriculture, new ways to tackle urban heat islands, or building coastal mangroves) and therefore lower the incentives to migrate overseas. Further, by enhancing resilience, adaptation aid could support economic growth, which may, in turn, reduce the incentives to emigrate, although we recognize the possibility of a non-linear relationship between economic growth and migration [32]. While most migration takes place within the country or spills over to neighboring countries [33–35], there is a perception (often fueled by populist parties) that a sizable percentage seeks to emigrate to the Global North. Thus, we also examine whether, in donor countries, the public willingness to support bilateral climate finance to specific overseas countries might also depend on their attitudes towards these countries.

Our analysis tests the argument that donor and recipient characteristics may also influence public support for overseas adaptation assistance. The political economy of aid literature has long stipulated that the nature of donor and recipient countries and their relations in different international forums affects who gives assistance, in what form, how much, to whom, and why [36–38]. Scholars have examined these issues in the context of foreign aid in totality as well as specific components such as humanitarian aid and environmental aid [39,40]. However, the extent to which different types of international linkages interact with specific country contexts to influence adaptation assistance has not been fully elaborated or explored in the literature. Acknowledging that recipient characteristics as well as economic and political linkages of the donor with recipient countries influence aid decisions, we explicitly build such expectations in our conjoint experiments.

Given the “aid fatigue” in many donor countries due to perceptions about the misuse of foreign aid in recipient countries [41], and the fear that adaptation assistance might crowd out domestic donor priorities, the first hypothesis captures a baseline expectation, similar to the Japan study, that public support for the policy package will diminish as the volume of bilateral aid increases. Hence:

Hypothesis 1: Support for the policy package will diminish when the volume of climate aid is high.

In the European context, immigration is a salient yet complex subject in an era of populism. There is a well-developed literature examining public support for immigrants in host countries [42–46], including immigrants with different characteristics such as skill level, ethnicity, religion, and gender. There is also an emerging literature examining public support for accepting climate migrants [47–49]. Scholars have examined how aid and migration are related [50–53], including how adaptation aid might influence migration [54]. The European Union’s generous aid package to Turkey and, more recently, to Tunisia suggests that industrialized countries are using foreign aid to hold back the inflow of migrants. Yet, it is not clear whether the public in donor countries sees the link between climate assistance and immigration. Arguably, given the populist backlash against immigration, the baseline expectation would be that the public in the Global North is less likely to support a policy package as the number of climate migrants increases. Hence:

Hypothesis 2: Support for the policy package will be lower when the number of climate migrants to be accepted from this overseas country is high.

Hypotheses 3 and 4 aim to elaborate the logic of how strategic linkage of issues at the international level may influence domestic support for different forms of climate adaptation assistance. Concerning bilateral aid, scholars note that donors have multiple motivations, including humanitarian, economic, and political. Specifically, countries might value their international standing in global forums and reward countries that vote alongside them in these venues [55]. Scholars note these sorts of “vote buying” in the context of the UN General Assembly [56–58], the UN Security Council [59,60], as well as more specialized forums such as the International Whaling Commission [61]. While such strategic linkage between aid and support at international forums takes place at the intergovernmental level, it is less clear how such linkages at the global level might impact public support for adaptation assistance. Theoretically, we elaborate on approaches that emphasize the relevance of public opinion for development aid policies and multilateral climate finance [24,25,62] to explore if the public responds to strategic issue linkages when expressing support for bilateral adaptation packages. More specifically, the conjoint experiment tests for such linkages in the context of the UN Security Council, given that Switzerland recently became a non-permanent member of the Council, and its role as such is relatively salient in the public’s view [63,64]. Thus, we hypothesize:

Hypothesis 3: Support for the policy package will be higher for countries that vote with Switzerland in the UN Security Council.

Trade relations constitute another international factor that could affect public support in donor countries for climate assistance packages. Some research suggests that trade displaces aid, while others find that donors provide aid to countries with whom they have more trading relationships [24,36,37,65]. Bilateral trade has furthermore been deployed strategically to gain traction on other international priorities such as human rights, labor rights, or the environment, and more recently on climate finance [61,66,67]. In international environmental politics, issue linkage with trade regimes has served to incentivize international agreements and treaty ratification [68] and in supporting domestic commitments for climate mitigation [69]. Uji et al. tested the impact of Japan’s imports and exports with an overseas country on support for adaptation assistance, capturing the potential relevance of domestic distributional implications [26]. Here we investigate whether the public view of linkages between international trade relationships and aid, rather than distributional implications, influence public support for adaptation packages. We hypothesize:

Hypothesis 4: Support for the policy package will be higher for countries that trade more with Switzerland.

The elaboration of H3 and H4 furthermore allows us to explore in greater depth, both theoretically and empirically, to what extent the linkage between climate aid and issues such as trade and support in UN forums might have broader repercussions for the level of public support through conditional effect on other relevant attributes examined in this study.

Hypothesis 5, in turn, explores the relevance of country characteristics related to migration patterns, such as geography, on public support for adaptation packages. Public support for foreign aid is influenced by the geographical location of the recipient country [56,57,70]. Examining the relevance of distance or proximity to Switzerland on respondents’ support for policy packages may be reflective of the European context at large, given the high and divisive attention placed on immigration waves from relatively close destinations such as North Africa and Syria. Thus, unlike respondents in other contexts such as Japan, where migration is not a salient issue, respondents might fear larger (climate) migration from a country that is proximate to Switzerland. Second, such risk perceptions might be stronger if the donor country has previously experienced high immigration levels from specific overseas countries. In recent years, Southern and Western Europe – including Switzerland – have witnessed a surge in immigration from African and Middle Eastern countries. Scholars note the emergence of bias and specific negative frames for specific migrant communities [71]. Research has furthermore revealed a tendency to instrumentalize and politicize immigration from these countries by populist right-wing parties [29]. Thus, it is important to recognize that, sometimes, race, ethnicity, and geography overlap. Support for adaptation assistance to specific countries might also be influenced by such implicit or explicit political, cultural, or racial biases [34,49].

To unpack these related issues, our survey instrument features four potential recipient countries that differ in terms of their distance from Switzerland and cultural characteristics such as religion and language spoken within each region. We chose Algeria, Kenya, Bangladesh, and the Philippines for several reasons. The countries in our study are located either in Asia or in Africa. They show high levels of vulnerability to climate change and have experienced extreme weather events. Their geographical distance to Switzerland varies, and so does the historical and current presence of these migrant communities from these countries. We selected two countries in Asia and Africa that vary in racial and cultural characteristics such as language, religion, and colonial history. Referencing actual rather than hypothetical countries is intended to simulate a more realistic context, to which respondents could relate more directly. Based on the preceding discussion, we hypothesize:

Hypothesis 5: Support for the policy package will be lower for countries located in closer proximity (Algeria and Kenya) as opposed to a far-off continent (Bangladesh and Philippines) in reference to Switzerland.

H5 suggests that we may expect context-specific dynamics to influence support for climate adaptation packages, reinforcing the value of the comparative perspective. The relevance of geographical regions and distance is likely to reflect the underlying politics of migration and vulnerability, as well as cultural and historical legacies associated, for instance, with colonialism. The inclusion of countries with different cultural and historical backgrounds within each region further allows us to explore indirectly if cultural biases related to religion and language, for instance, could be detected with respect to public support for adaptation packages. This is to our knowledge one of the first studies to explore the underlying politics that may influence public support in the Global North for climate assistance for overseas countries located both across and within continents.

Finally, the last two hypotheses, consider how different types of extreme weather events might influence public support for adaptation assistance, again with attention to the Swiss context as a donor country. Given that important regions and sectors in Switzerland, including water, tourism, agriculture, and the Alpine regions more generally, are highly vulnerable to climate change, the Swiss public might be more sympathetic to countries that experience similar extreme weather events (floods and droughts as opposed to cyclones or sea level rise). Swiss residents may relate more directly to the consequences of such events and the resulting need for adaptation. Alternatively, the intensity of other extreme weather (such as cyclones and floods) tends to be captured dramatically in the media, which might enhance public support for adaptation assistance to overseas countries [34,48,49]. Such a portrayal can evoke empathy for countries that have contributed little to climate change but whose human security and economies are threatened by sudden-onset events over which they have little control. There may be a perception, because of media attention, that sudden-offset severe events are likely to bring about more severe damage, human displacement, and suffering and thus require immediate adaptation assistance. We thus distinguish between a “linked fate” hypothesis, referring to shared experience with climatic weather events (Hypothesis 5a), and a “sudden-onset events” hypothesis related to their anticipated high impact and salience (Hypothesis 5b).

Hypothesis 6a: Support for the policy package will be higher for countries that experience floods and droughts as opposed to sea-level rise and cyclones.

Hypothesis 6b: Support for the policy package will be higher for countries that experience sudden-onset events, such as floods and cyclones as opposed to slow-onset events such as sea level rise and droughts.

## Case selection and model

In terms of case selection, we focus on Switzerland because it has a consistent record of implementing climate mitigation policy commitments under the UNFCCC [72–74]. The most recent Climate and Innovation Act, known also as the Net-Zero Climate Law, sets intermediary objectives and means for reaching net-zero emissions. The government proposal was supported by popular vote in June 2023 with 59.1% of the votes. Broadly, opinion polls report a high level of awareness of vulnerability to climate change as well [75]. This reflects, in large part, the ecological sensitivity of the country’s Alpine region (including the issue of receding glaciers), its importance for core sectors such as tourism, agriculture, water, and energy, as well as citizens’ identification with and attachment to the Alpine landscape. Thus, Swiss respondents are likely to have a high appreciation for the need to adapt, even as the country seeks to mitigate global climate change.

Furthermore, because there is a strong element of direct democracy in Switzerland, where voters are called to express their opinion on specific policies multiple times every year, the use of surveys is highly appropriate in the Swiss context to assess public support for a given policy [76]. The results can inform policymakers about specific frames or features that drive individuals’ support for overseas climate adaptation assistance.

The Swiss context also captures the broader trends in European and global politics to both the unfolding trajectory of climate change and the rise of right-wing parties. Following the highly salient heatwave of 2003, Europe has experienced a steady increase in average temperature, droughts, and sudden onsets of floods and wildfires. Such events are widely covered in the media. At the same time, with the rise of right-wing parties, issues such as climate change, development aid, and migration have become more contentious and salient. By probing public opinion on the question of overseas adaptation assistance in the case of Switzerland, this paper seeks to provide new empirical evidence on the interface of domestic public opinion with climate aid and immigration.

Switzerland constitutes a strong candidate for replicating the Japan study by Uji et al. [26]. While immigration is not a salient issue in Japan, it is a highly salient one in Switzerland, due to the politicization and instrumentalization by right-wing parties [27,28,29]. In addition, the rising immigration flows have motivated European countries to offer aid to overseas countries to stem immigration [30] – a strategy officially acknowledged as one of the objectives of Switzerland’s development assistance by the Swiss government [77]. The financing of development aid, moreover, has become a contentious issue in Swiss politics – with drastic cuts proposed and supported by right and center-right parties in recent years [78]. These characteristics suggest that both adaptation aid and immigration are more salient and contested in the Swiss context as opposed to Japan.

Replicating the study in the Swiss context and comparing results across these cases is important for several reasons. First, in and of itself, the replication of the methodology in another donor country expands the comparative understanding of the factors that shape public support for policies that increase adaptation aid and acceptance of climate migrants, and the overall external validity of the results. While replicability is a core tenet of rigorous and impactful research, it tends to be practiced much more readily in the natural sciences, compared to social sciences. This is often a consequence of data availability, concerns about comparability of units of analysis, and discounting the practical value of such research, unlike in life sciences where replication is a core and necessary element of practical applications. We contend that to improve policy understanding of the politics that could unlock support for increased adaptation assistance, systematic examination of such factors across different donor countries is necessary.

Second, not only do Japan and Switzerland differ in key respects when it comes to the salience and politicization of migration and aid, but also as previously mentioned demographic and political situations observed in Switzerland are part of a broader European trend. As such, while cautious of the generalizability of results beyond the Swiss context, this comparison may offer a first understanding of preference formation in contexts where immigration and aid are highly salient and politicized topics.

## Methods

Empirically, we examine public support for bilateral climate adaptation assistance via a conjoint experiment nested in a survey experiment in Switzerland. As public policy is at least partly responsive to public opinion [79], surveys are an important tool to assess preferences about existing or proposed policies. Specifically, a conjoint experiment, contrary to other experimental designs, allows for a concurrent assessment of the causal effect of different policy attributes on public support for climate adaptation policies. Such conjoint experiments reduce social desirability bias because respondents can implicitly trade off attributes against each other. This makes them particularly suited to studying the drivers of policy preferences. Validation efforts of survey-experimental conjoint methods have shown that this experimental approach closely replicates the outcomes in popular votes [80].

If individuals are boundedly rational, their policy preferences might be influenced by how policies are framed. This is why we embedded the conjoint in a survey experiment where respondents are randomly assigned to two treatment frames that highlight aid and migration policies of peer countries as well Switzerland’s past policies on these subjects.

Survey experiments are sometimes criticized for their drawbacks in terms of external validity, they only offer a snapshot of a given case at one point in time, and they may not always mirror real-world situations. The conjoint design of our experiment and its setting mitigate some of the above concerns. On the one hand, conjoint experiments are thought to have a better generalization potential compared to other survey experiments because they better approximate real-world situations where individuals are asked to evaluate policies with multiple attributes [81]. Such evaluation exercise is, moreover, quite common in the semi-direct democratic context of Switzerland, where individuals often evaluate policies and their trade-offs when called to vote upon them [76]. Lastly, the method lends itself to rigorous replication and comparative understanding of the politics of climate aid, in this case concerning a prior analysis of Japan.

### Sampling strategy, sample, and survey administration

We administered an online survey-embedded conjoint experiment to a sample of 876 Swiss residents aged 18 and above. Respondents were drawn from a *Qualtrics* panel. We established quotas on gender, age, and spoken language to ensure the sample was representative of the Swiss population, which resulted in a representative sample of 876 respondents. *Qualtrics XM* ran this survey between July 4 and July 19, 2023, in the three Swiss official languages (German, French, and Italian).

Internet panels have their drawbacks, compared to population-based probability sampling, particularly regarding their representativeness of older age groups of society with lower internet access [82]. However, studies have shown that Qualtrics panels perform well when compared to population-based random samples and better than those of other providers [83,84]. Furthermore, the possibility of setting quotas – and their use – can mitigate these concerns. The data in our analysis is representative across key dimensions of the Swiss population.

After Human Subject Approval was obtained by the authors, the experiment was pre-registered (https://osf.io/wahxb?view_only=73e3c88760ed478e87c90a4fcc86add2). Moreover, given that the survey was to be administered in three different languages of Switzerland (German, French, and Italian), authors informally pre-tested the questionnaire with native speakers before the official launch to make sure that the questions were written clearly and understood by respondents across linguist groups.

Informed consent, anonymity, and confidentiality: Informed consent was sought at the beginning of the survey. Each participant had to read the informed consent statement and could decide whether to accept and agree to participate or exit the survey. In the informed consent statement, participants were informed of the purely scientific purpose of the paper and the lack of commercial and government-related purposes. Anonymity and confidentiality guarantee related to data collection, storage, and publication were explicitly stated in the informed consent statement. The statement also included an email address at which participants could direct their concerns, which included the name of one of the authors’ institutions. The objective of the study was explained to participants, who have been informed at the consent stage of this process that the authors intended to study public opinion on climate change. They were also informed of the length of the survey. The informed consent statement is available in the survey instrument in Supporting information S1 File.

### Conjoint experimental design

In the design of our experiment, we ask respondents to compare two overseas adaptation assistance packages in different countries. In each package, respondents consider two forms of assistance – aid and immigration along with different types of connections the recipient country has with Switzerland. The ability of conjoint experiments to capture such multi-dimensionality makes them better able to mirror real-life situations. Conjoint experiment design further lessens the risk of social desirability bias because respondents can implicitly trade off one attribute against another [85].

We draw broadly on the approach implemented by Uji et al. [26] in the context of Japan. First, all respondents initially read a paragraph laying out the causes of climate change, explaining the role played by industrialized countries, including Switzerland, in the accumulation of greenhouse gas (GHG) emissions, and how developing countries facing extreme weather events can be supported, either by providing adaptation aid or by accepting climate migrants. The possibility that Switzerland is expected as an industrialized country to provide adaptation assistance was presented in a neutral, non-normative tone, to decrease the risk of social desirability bias. The introductory paragraph was followed by a question that served as an attention check.

In the experimental phase of the survey, we randomly assigned participants to one of the three groups: a control group and two treatment groups. The objective was to assess if providing information about peers or what Switzerland has done in the past regarding aid or immigration, might frame the conjoint experiment for respondents in different ways. Data on Switzerland’s overall acceptance of migrants was obtained from the UNHCR Refugee Data Finder, while the data on bilateral climate aid comes from the OECD Development and Cooperation Profiles.

The first treatment group (peer benchmarking) received information about annual levels of bilateral climate aid and refugee acceptance in OECD countries. The second treatment group (self-benchmarking) received similar information about Switzerland. The third group, serving as the control or reference category, received no benchmarking information.

After reading the benchmarking information, respondents were provided with instructions about the task they were about to complete and were informed they were about to be shown different hypothetical policies that the Swiss government could adopt to support developing countries in adapting to climate change.

Participants were asked to compare two policy packages, each with six dimensions. Then they were asked to select the one they would recommend the Swiss government to adopt. They repeated the choice task six times. This type of paired-options, forced-choice conjoint design is believed to promote higher engagement of respondents with the task at hand [86].

The six attributes in the conjoint are: (1) the recipient developing country (Algeria, Kenya, Bangladesh, Philippines); (2) the level of climate aid Switzerland will provide to the recipient country per year (0 CHF, 30 million CHF, 60 million CHF, 90 million CHF, 120 million CHF); (3) the number of immigrants from the recipient country Switzerland will accept per year (0, 250, 500, 750, 1,000, 1,250); (4) the type of extreme weather event that the recipient country experiences (drought, sea level rise, flood, cyclones); (5) the volume of trade between Switzerland and the recipient country (0, 500 million, 100 million); (6) the percentage of times the recipient country voted with Switzerland at the UNSC (0%, 40%, 80%).

To present respondents with credible information, we set the levels of the trade attribute using official data available on the website of the Swiss Federal Department of Foreign Affairs on the trade volume between Switzerland and each one of the recipient countries (https://www.eda.admin.ch/, accessed 2003). Table 1 displays attributes, their labels in the survey, and the possible levels.

**Table 1:** Conjoint experiment attributes and levels.

Attributes	Labels	Levels
Recipient country	Recipient developing country	Bangladesh/Philippines/Algeria/Kenya
Level of climate immigration	Number of climate migrants to accept from this country per year	0/250/500/750/1,000/1,250
Level of climate aid	Climate aid to give to this country (CHF) per year	0/30 million/60 million/90 million/120 million
Trade volume	Value of Swiss trade with this country (CHF)	0/500 million/1,000 million
Extreme weather event	Extreme weather event	Drought/Sea level rise/Floods/Cyclones
UNSC voting alignment	Percentage of this country’s votes in line with Switzerland’s position at the UN Security Council	0%/40%/80%

The value any attribute took randomly varied within and across choice tasks. The only restriction to randomization was that the policies constituting a pair within each choice task always referred to two different countries, to avoid instances in which a respondent would be asked to choose between policy packages referring to the same country.

**Fig 1:**
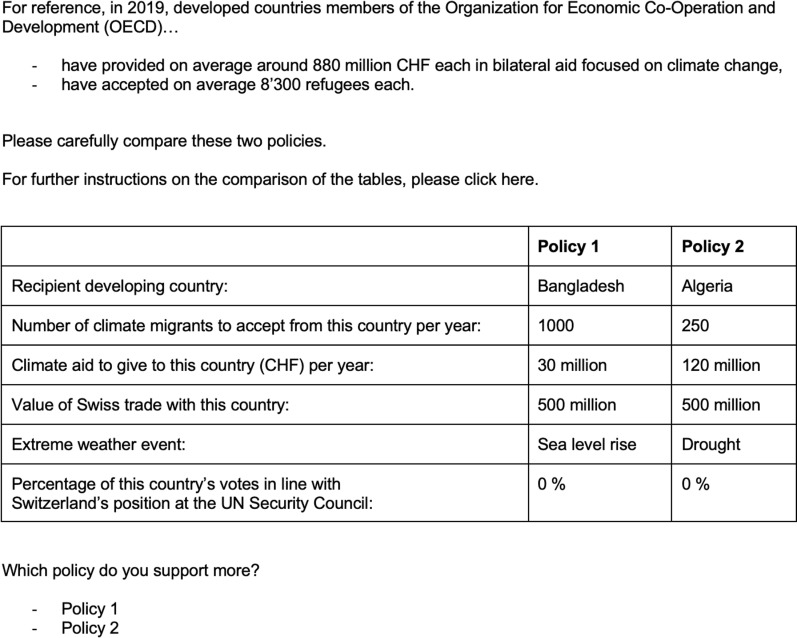
Example: choice task with benchmarking on OECD countries’ policy.

Because we want to understand public support for bilateral adaptation packages, the recipient country always appeared first, followed by two forms of adaptation assistance, aid level and immigration level (the order in which these attributes appeared in the conjoint table varied randomly between positions 2 and 3, across respondents) and, lastly, recipient country characteristics such as extreme weather event, trade volume, and UNSC vote convergence (the order in which these attributes appeared in the conjoint table varied randomly between position 4, 5 and 6, across respondents). For any respondent, the order of the attributes that appeared in the conjoint table was kept constant across six iterations, to reduce cognitive burden (Fig 1).

Following the completion of the six choice tasks, respondents were asked to answer questions to assess their personal and/or cultural beliefs that may influence their policy preferences. Given the high level of contention around immigration in Swiss politics, we posed questions about respondents’ beliefs about whether linguistic and cultural proximity might help immigrants integrate with Swiss society, whether development aid reduces poverty, and the extent to which they feel pride toward Swiss history and culture.

Specifically, respondents were asked to indicate the extent to which they agreed or disagreed with the following statements: “Immigrants from countries whose languages are similar to those spoken in Switzerland will integrate more easily in Switzerland”; “Immigrants from countries that are culturally similar to Switzerland will integrate more easily in Switzerland”; “I am proud of Swiss culture and history”; “I believe that development aid reduces poverty”.

The survey included questions aimed at measuring standard socio-demographic variables (gender, age, area of residence, education, occupation, economic sector, income, and political ideology) which can independently influence the respondents’ support for the overseas adaptation package. The full questionnaire is available in Supporting information S1 File. Replication data is available at the Harvard Dataverse: https://dataverse.harvard.edu/dataset.xhtml?persistentId=doi%3A10.7910%2FDVN%2F3YWEEV&version=DRAFT

## Results and discussion

We use marginal means (MMs; [87]) analysis to estimate the respondents’ favorability for different policy attributes and levels among a given attribute. Conventional measures of interaction effects such as Average Marginal Component Effects (AMCEs) require the arbitrary choice of a baseline category, which can affect the directionality, size, and significance of coefficients. In contrast, MMs summarize outcomes by averaging predicted probabilities across all levels of attribute or covariate combinations. By averaging these predicted probabilities, MMs provide an intuitive and comprehensive summary of the favorability of the policy package with a given attribute level [87]. Because of these advantages, MMs have become an established method for conjoint survey experiments. Thus, we interpret marginal means from a forced-choice conjoint survey experiment as the favorability of a policy package with a given attribute level to gauge public support.

**Fig 2:**
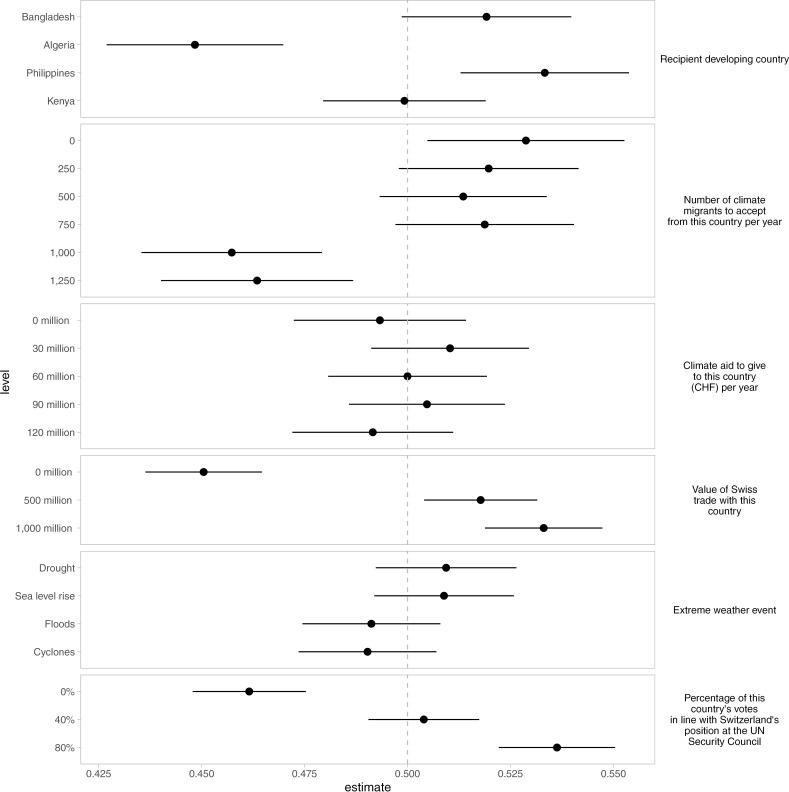
Baseline favorability for the overseas climate adaptation assistance policy package.

Fig 2 presents MMs, whereby the vertical dashed line represents the 50% (0.5) threshold. Coefficients higher than 0.5 reflect that a policy proposal with the respective attribute level increases the favorability of a climate adaptation policy (as long as the confidence interval around the point estimate stays on the right of the 0.5 level), while coefficients lower than the threshold reflect lower favorability (as along the confidence band around the point estimate stays on the left of the 0.5 level). We discuss the results along the different dimensions of our argument and specific hypotheses. Descriptive statistics are presented in Supporting information S2 Fig.

### Limited unconditional support for climate aid or climate migration

We found no statistically significant effects for adaptation packages at any level of climate aid. This is surprising because supporting Global South countries in climate adaptation is now an important issue in global policy conversations. Previously, Global North countries had pledged annual climate aid to the tune of US$100 billion. This was revised upwards to US$300 billion at the 29^th^ Conference of Party meeting in Baku (although this is far below US$1.3 trillion demanded by Global South countries). Of course, how much of the aid will be devoted to climate adaptation remains unclear, yet climate aid has gained more prominence on the world agenda. We do not find Swiss respondents expressing a preference for policy packages with high or low levels of adaptation aid (Hypothesis 1 is not supported).

While the limited preference of respondents for adaptation aid is consistent with the findings by Gampfer et al. [25] in other country contexts, it is somewhat surprising in the Swiss context, as Switzerland has a reputation for being one of the first Global North countries to provide adaptation aid [88]. According to the latest available data from the OECD Development Assistance Committee (DAC), Switzerland increased climate aid as a percentage of bilateral aid to 23% in 2020–21 compared to 19.5% the previous year, with a greater share devoted to adaptation assistance compared to mitigation [89]. However, despite its stronger emphasis on adaptation compared to other OECD countries, Switzerland still lags in terms of the share of bilateral aid directed to climate issues compared to the OECD average [89]. More recently, the Swiss Agency for Development and Cooperation and the State Secretariat for Migration have established a new program for climate change and migration “to create better prospects” for youth in North and West Africa and establish safeguards against youth trafficking and abuse [90]. Such policy initiatives are illustrative of the concern about proximity and the political salience of the region, as well as linkages between climate adaptation, development prospects, and migration. Such concerns, however, are not reflected at the level of public opinion according to the results of our conjoint survey experiment.

Migration is also a form of adaptation: by accepting climate migrants, Switzerland could support overseas climate adaptation. However, we find that Swiss respondents favor policy packages with zero immigration and disfavor high levels of immigration (1,000, 1,250). This likely reflects the populist backlash against immigration in many European countries, including Switzerland (Hypothesis 2 is supported).

Attributes that capture exposure to different types of extreme weather events similarly do not have a significant impact on public support for policy packages (Hypotheses H6a and H6b are not supported). This is somewhat surprising for Switzerland, given the high awareness about vulnerability to weather extremes domestically. The above discussion is summarized in Fig 2. below (and Supporting information S3 Table)

### Issue linkage with trade and UN voting alignment

**Fig 3:**
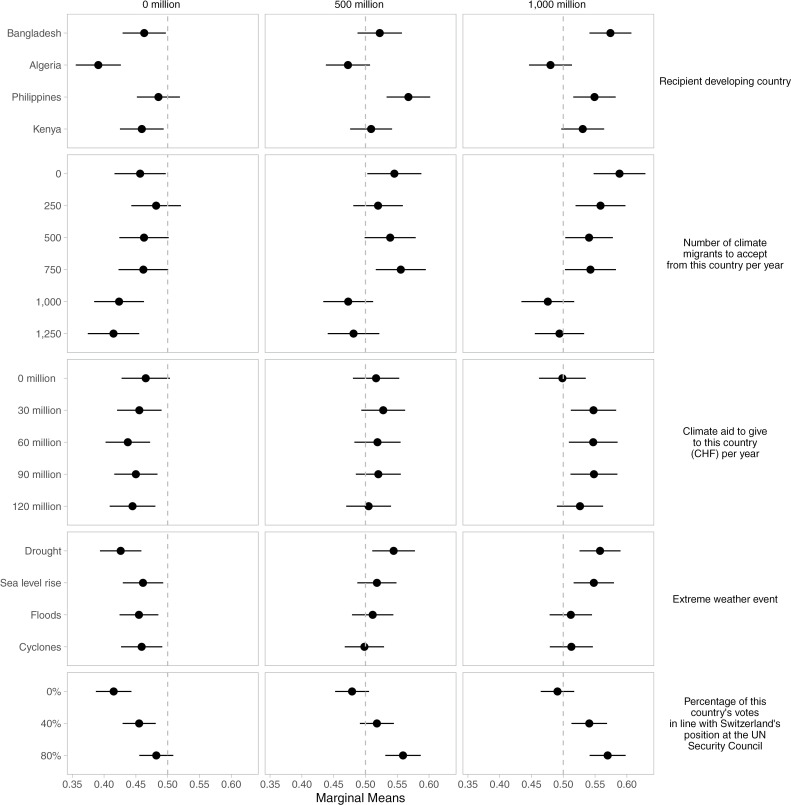
Trade conditions the effect of the other five conjoint attributes on the favorability of policy packages by respondents.

The results of the survey experiment show that Swiss respondents prefer adaptation policy packages for trading partners and for countries that vote with Switzerland in the UNSC. As shown in Fig 3, 80% alignment of votes with the Swiss position in the UN Security Council increases the favorability of policy packages, while the 0% alignment scenario reduces support, with the mid-range (40%) being statistically non-significant (Hypothesis 3 is supported). Similarly, respondents favor policy packages for countries with medium and high levels of trade with Switzerland, while support is significantly reduced for countries with zero volume of trade (Hypothesis 4 is supported). These findings suggest that respondents tend to view climate adaptation assistance as part of the broader spectrum of international economic and policy engagement between Switzerland and developing countries – that is, they are not compartmentalizing climate policy.

The significant impact of the high share of UN vote alignment and high volumes of trade on public willingness to support adaptation packages to developing countries suggests that the public may view such countries as strategic allies in international affairs more broadly and with respect to climate adaptation more specifically. These findings prompted us to further explore how such two-level issue-linkage might condition the effects of other conjoint attributes. Importantly, similar results were found in the case of Japan for vote alignment in the UN General Assembly, and countries to which Japan has a high volume of exports. The significance of these factors across the different political contexts of two donor countries, in terms of trading partners, geographies, and policies related to climate, migration, and economic development, suggests that two-level issue linkage deserves further exploration with respect to public motivation to support for increasing adaptation assistance.

**Fig 4:**
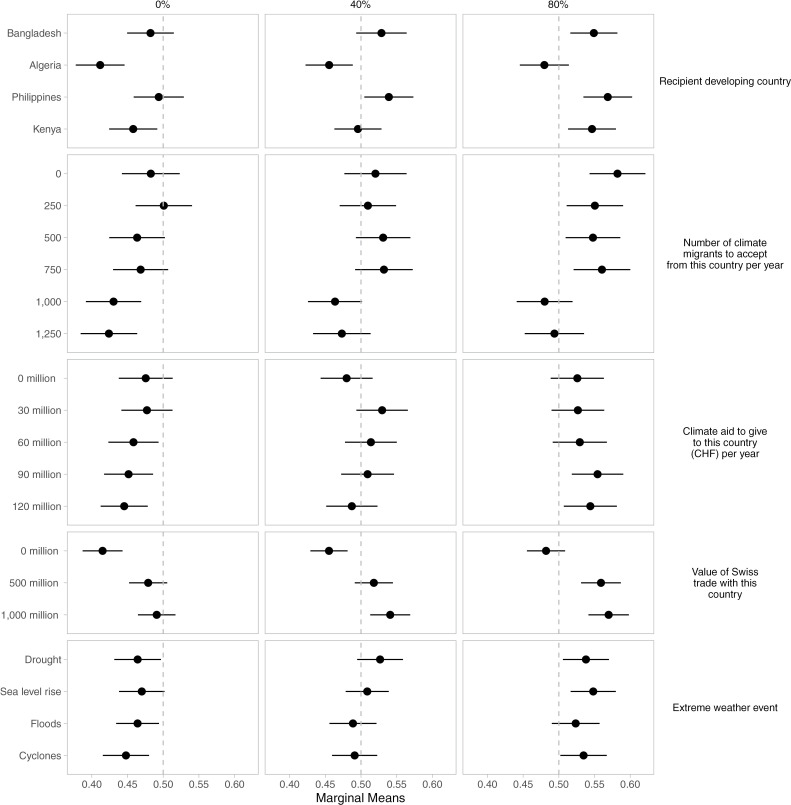
UN Votes condition the effect of the other climate adaptation assistance conjoint attributes on the favorability of policy packages by respondents.

Notably, the results on the conditional effects of trade volume and vote alignment in the UN Security Council provide further evidence of the significance of trade relations and UN Security Council position alignment for respondents’ preference of policy packages. As shown in Figs 3 and 4, the highest bilateral trade volume (1,000 million) is associated with a positive shift in respondents’ favorability of substantial bilateral aid packages, ranging from 30mn/year to 90mn/year. Similarly, respondents tend to favor packages with low and medium levels of climate migration (250 to 750 per year). By contrast, when there are no trading relationships, respondents are indifferent about policy packages containing 0 million of climate aid (the confidence interval overlaps the dashed line at 0.5, indicating indifference). Policy packages with positive levels of climate aid reduce respondents’ favorability towards policy packages. These results are broadly robust to a different modeling strategy, specifically, nonparametric ANOVA regression [91], allowing the selection of the most important interaction effects between the different conjoint attribute levels (Supporting information S17–S19 Tables, S20 Fig, S21–S22 Tables). These results show that policy packages in which countries do not trade with Switzerland decrease the policy package’s favorability in almost all treatment combinations of the attribute levels in the conjoint survey experiment.

Fig 4 further shows that the conditional effect of an 80% alignment of votes with the Swiss position at the UN Security Council increases the favorability of policy proposals with the highest range of bilateral climate aid packages (90mn/year and 120mn/year), as well as for packages with low- and medium-level migration (250–750 persons/year). Significantly, the interaction with high levels of UN Security Council alignment and trade volumes makes respondents more likely to favor a broader range of countries (Philippines, Bangladesh, and Kenya).

By contrast, the conditional effect of 0% alignment in the UN Security Council is associated with negative or non-significant favorability for packages. These results strengthen our confidence in the robustness of our findings concerning Hypotheses 3 and 4 on how linking issues such as trade and foreign policy positions is likely to strengthen the support for policy packages of climate adaptation assistance. Yet, if such issue linkage strategies were to be used for increasing adaptation aid and willingness to accept climate migrants, their consequences for environmental equity might also be negative, as aid might not go to countries that are the most vulnerable and least resilient, but to those with a strong competitive advantage and strong international trade engagement and institutions.

### Geographic regions and country perception

The results of our baseline model in Fig 2 (which pools respondents across control and treatment groups) reveal notable differences in the favorability of climate aid and climate migration policy packages across recipient countries. Swiss respondents tend to favor policy packages for the Philippines, while they tend to disfavor policy packages for Algeria. We find indifference towards Bangladesh and Kenya. Stronger positive favorability for Bangladesh policy packages and in some instances for Kenya policy packages becomes evident in conditional effects models, which we discuss later. Results for Hypothesis 5 are thus mixed. For reference, in the Japan study, Uji et al. [26] find partial support that the public favors climate adaptation assistance to neighboring Asian countries. Specifically, they found that Japanese respondents support policy packages to Bangladesh (but not to Cambodia) in relation to Tanzania (Africa) or Peru (Latin America).

What might account for such variation among Swiss respondents in policy package favorability across recipient countries? As previously hypothesized, some respondents might associate the geographical location of countries with the likelihood of increased migration. For example, migrants from a geographically closer country may face lower costs of moving to Europe and may have access to more informal routes to reach a country such as Switzerland. Consistent with Hypothesis 5, we do find that Algeria – the country closest geographically to Switzerland – as the recipient country has a significant negative effect on policy favorability. Although the Algerian community in itself is not very strongly present in Switzerland (4,152 Algerian nationals, [92]), the total number of foreign citizens from the Maghreb (i.e., Tunisia, Morocco, and Algeria) is quite significant for Switzerland (around 20,000 individuals, [92]). Hence, historical immigration inflows, coupled with the proximity of Algeria to Switzerland, could partially explain this result.

Furthermore, in the European context, immigration, particularly from Africa and the Middle East, has become a politically salient issue. Media stories about boat crossings from North Africa are a regular feature in the news cycle, which can shape public views in this respect [93]. Studies conducted in West European countries have further suggested that immigrants from North Africa became subject to security- and cultural-threat frames in European media [71]. Moreover, France is a former colonial power in North Africa, which has shaped both migration patterns but also prejudice vis-à-vis migrants. These factors could influence perceptions about migration from countries such as Algeria, given the extensive land borders and linguistic connection between France and Switzerland.

The favorability of policy packages containing immigration in host countries is sometimes influenced by the perceived demographic characteristics of immigrants, such as gender, linguistic fluency, and skill level [94]. Swiss citizens potentially associate immigrants from specific countries with certain types of demographic characteristics, and this influences their favorability for overseas climate policies.

At the same time, we find that Swiss respondents are indifferent regarding Kenya – also an African country and more proximate to Switzerland compared to Bangladesh and the Philippines. This could be because immigration from Kenya specifically is neither salient nor politically instrumentalized. The Kenyan community in Switzerland is quite small, with 1,853 citizens residing in Switzerland in 2022 [92], and these factors might explain the overall indifference of the respondents.

Similarly, the South and Southeast Asia region might not be directly associated in the public mind with waves of migration as they do not receive much media attention. Moreover, unlike North Africa, the substantial distance from Asia implies a lower likelihood of climate migrants seeking shelter in Switzerland. Thus, reference to these countries does not diminish the favorability of climate policy packages. Yet, our results are mixed in this regard, as respondents are indifferent to Bangladesh but prefer policies aimed at the Philippines. The consistent preference for the Philippines thus deserves additional exploration. Filipino residents in Switzerland do not constitute a large community (6,423 individuals, [92]). Still, similarly to Algerians, they are more present than Bangladeshi and Kenyan citizens (around 1,704 and 1,853 respectively, [92]). Whether Swiss residents hold particularly positive social representations of the Filipino community, and whether this explains our results, however, should be researched further.

Because of the multiple factors encompassed by geographical location and the absence of a direct test of the proposed mechanisms that would allow for disentangling them, additional studies are needed to understand what specific features of the recipient country drive citizens’ favourability of policy packages. Interestingly, however, our results suggest that the identity of the recipient country influences policy favorability only in specific subsets of the sample (Supporting information S8 Fig, S9 Table, S10 Fig, S11 Table, S12 Fig, S13 Table). Indeed, the significant positive effect for the Philippines and the negative effect of Algeria disappears amongst young respondents (18–34 years old), amongst highly educated ones, and respondents with medium to high incomes. In other words, the results are insignificant for each of the four recipient countries in these sample sub-groups. This may imply that racial and cultural prejudices are lower among the younger generation and demographics with higher education and income (Supporting information S4 Fig, S5 Table, S6 Fig, S7 Table, S8 Fig, S9 Table).

At the same time, however, the position of respondents on the political spectrum (Supporting information S23 Fig, S24 Table), their beliefs about integration (Supporting Information S12 Fig, S13 Table), and their pride towards Switzerland do not significantly alter our main results in this respect (Supporting Information S14 Fig, S15 Table).

### Benchmarking and support for adaptation assistance

Recall that the conjoint experiment is nested within a framing experiment. Might the respondents’ favorability of aid or immigration be conditioned by benchmarking with other OECD countries (Treatment 1), or with Switzerland’s existing policies (Treatment 2)? After all, the general public may not have deep knowledge about actual aid and immigration levels within Switzerland or in its peer countries. Thus, providing context might reduce the knowledge gap and shape the policy preferences of respondents. We find no significant differences across treatment groups. In further analysis, the interaction of aid and immigration with treatment dummies does not produce statistically significant results (Supporting Information S16 Fig). This is broadly consistent with Uji et al. [26] which reports that benchmarking with respect to past Japanese policy and peer G7 countries did not produce statistically significant effects.

### Conditional effects of political ideology and cultural beliefs

Finally, to further explore the robustness of the main findings of our experiment, we also explored the conditional effect of political ideology and cultural beliefs as factors likely to influence the favorability of policy packages, given the highly contentious immigration and climate policy debates. Studies of climate politics in Switzerland note the consistent divide between right-wing and left-wing coalitions on climate policies [72–74,95]. Research on public opinion and foreign aid has similarly emphasized the variation across the left-right political ideology spectrum [23,24,46]. While conservatives tend to oppose migration, we do not find political ideology to condition the favorability of policy packages either for the aid or immigration dimensions of the policy package (Supporting Information S23 Fig, S24 Table).

Finally, we examine how individual beliefs might condition preferences for policy packages, even after controlling for ideology. Consider the interaction effects of cultural views based on consideration of language affinities (Supporting Information S10 Fig, S11 Table), cultural affinities (Supporting Information S12 Fig, S13 Table), and expressions of pride for Swiss history and culture with the baseline model (Supporting Information S14 Fig, S15 Table). All these factors are important in the contemporary discussion on populism. Much to our surprise, these interaction effects did not influence results in a statistically significant manner. Divergent views on the utility of development aid as a policy instrument (or lack thereof) similarly did not significantly affect our results.

## Conclusion

This paper makes several contributions to the study of climate politics. We join the growing movement that links climate policy to broader policy debates on immigration, foreign aid, and trade. Substantively, this study provides important insights into how Swiss respondents view the subject of climate aid and immigration in the context of specific developing countries. It reveals that the favorability for policy packages that substantially increase climate adaptation aid correlates positively and consistently with recipient countries’ trade volumes and voting alignment with the Swiss position in international forums such as the UN Security Council. Respondents consider such issue linkages in expressing their favorability for adaptation assistance. The favorability of policy packages with climate adaptation aid and accepting migrants increases as countries trade more with Switzerland and vote alongside the Swiss position in the UN Security Council. High levels of trade and alignment of votes in the UN Security Council with Switzerland increase the favorability of policy packages with ambitious aid levels and some level of climate migrant acceptance. While Switzerland maintains its neutrality in global politics, it is notable that public respondents (as opposed to policy elites according to standard expectations of the literature) regard countries that vote alongside Switzerland at the UN Security Council as more desirable partners for climate adaptation assistance.

These results resonate with theories on development aid and issue linkage to gain traction on other international priorities. They furthermore suggest that establishing linkages with other issues (such as trade) and international regimes (such as international security and potentially other domains) could strengthen awareness domestically and public opinion in favor of increasing climate adaptation assistance. To our knowledge, the politics of linking adaptation finance with other international issues, such as trade and UN-level cooperation, has not been previously explored in the literature. Comparable findings in the case of Japan for UN General Assembly vote alignment and countries receiving Japanese exports [26] further imply broader generalizability of the relevance of such issue linkage for directing climate adaptation assistance beyond the Swiss context. Such political dynamics and their implication can be explored with a broader comparative perspective, including for traditionally large donors such as the United States and countries in the European Union.

Another significant result is the evidence of diminished support for the assistance package that is directed to Algeria, while positive support is provided for packages directed to the Philippines. This suggests that the political and media representation and rhetoric aimed at North African countries might be influencing the perceptions of Swiss respondents. We are reluctant to frame this as purely religion-based discrimination bias because Swiss respondents do not reveal these negative preferences towards Bangladesh, another country with a similar background in terms of the prevalence of the Muslim religion, but with different geographic proximity and colonial past linked to continental European. Equally, despite cultural similarities between the Philippines and Kenya in terms of large shares of predominantly Christian population, we found support for the Philippines but a lack of support for Kenya, except for models with conditional effects of trade and UN voting. Thus, future research should explore the multidimensionality of factors that might guide public support for climate-induced migration and how this coheres with other drivers of migration, such as war and economic distress.

In the context of Switzerland, as in other industrialized countries, climate mitigation has dominated climate change politics. To the extent that adaptation is discussed [95,96], it is in the context of vulnerabilities at the domestic level, exemplified in the case of Switzerland by concerns about mountainous regions and vulnerable sectors, or concerning most vulnerable sections of society. Climate policy proposals have thus focused primarily on mitigation objectives under the overarching international treaties including the UNFCCC, the Kyoto Protocol, and the Paris Agreement [97]. Our study suggests that as a consequence of the traditional focus and contestation of climate mitigation, questions of adaptation aid and finance have fallen on the sidelines of public attention at best, as indicated by the insignificant results in the absence of linkage to trade and alignment of Security Council votes.

These findings are important, as they are likely indicative of a broader tendency of low public attention to international adaptation aid in industrialized countries. This raises questions about what kind of international platforms and domestic policies are needed to both increase the salience of adaptation aid and foster productive issue linkage for its ratcheting up. From a justice perspective, however, issue linkage could provide a double-edged response. Projecting a strategic view of climate adaptation aid and international trade, security, and other strategic priorities of donor countries may increase the public support for, and volume of, adaptation finance. Yet, this might not be necessarily good news for climate justice, as the direction of aid is more likely to carry the imprint of the economic and foreign policy interests of donors. The issue linkage perspective thus raises questions for further research on what kinds of linkage ought to be fostered across international regimes and norms of justice, equity, and humanitarianism, as means of amplifying the domestic resonance of adaptation aid policies.

Finally, while we focus on adaptation, we recognize that some effects of climate change (such as desertification, salination, or the loss of land to sea level rise) are particularly hard to adapt to [98]. Hence, the 2015 Paris Agreement officially recognized (Article 8) that the subject of Loss and Damage needs to be addressed separately from adaptation. Future work could explore whether the emergence of Loss and Damage as an issue in its own right might strengthen or crowd-out support for climate adaptation.

## Supporting information

S1 File Survey. (PDF)

S2 Fig Descriptive statistics. (PDF)

S3 Table Baseline estimates. (PDF)

S4 Fig Interaction with age. (PDF)

S5 Table Interaction with age. (PDF)

S6 Fig Interaction with education. (PDF)

S7 Table Interaction with education. (PDF)

S8 Fig Interaction with income. (PDF)

S9 Table Interaction with income. (PDF)

S10 Fig Interaction with immigrant language. (PDF)

S11 Table Interaction with immigrant language. (PDF)

S12 Fig Interaction with culture. (PDF)

S13 Table Interaction with culture. (PDF)

S14 Fig Interaction with Swiss pride. (PDF)

S15 Table Interaction with Swiss pride. (PDF)

S16 Fig Interaction with the experimental benchmarking. (PDF)

S17 Table Interaction the with experimental benchmarking. (PDF)

S18 Table Interaction with Swiss trade. (PDF)

S19 Table Interaction with UN Security Council votes. (PDF)

S20 Fig Average Marginal Interaction Effect (AMIE). (PDF)

S21 Table Fifty treatment combinations with the highest treatment effect. (PDF)

S22 Table Fifty treatment combinations with the lowest treatment effect. (PDF)

S23 Fig Interaction with the left-right political spectrum. (PDF)

S24 Table Interaction with the left-right political spectrum. (PDF)
