# Supplementary material for: International climate adaptation assistance: Assessing public support in Switzerland
Source: PLoS One. 2025 Feb 12;20(2):e0317344. doi: 10.1371/journal.pone.0317344 (PMC11819516; doi:10.1371/journal.pone.0317344)
Supplement: S1 File — (PDF) [file pone.0317344.s001.pdf]

S1 Survey.

1. Research Ethics and Adherence with the 2020 APSA's Principles and Guidance on Human Subject Research

The authors affirm that the manuscript adheres to 2020 APSA's Principles and Guidance on Human Subject Research.

IRB approval procedure: the study was submitted for approval to the IRB of the authors' institutions, and approval was obtained prior to the launch of the experiment.

Informed consent, anonymity, and confidentiality: Informed consent was sought at the beginning of the survey. Each participant had to read the informed consent statement and could decide whether to accept and agree to participate or exit the survey. In the informed consent statement, participants were explained the purely scientific purpose of the paper, and the lack of commercial and government-related purposes. Anonymity and confidentiality guarantees related to data collection, storage and publication were explicitly stated in the informed consent statement. The statement also included an email address at which participants could direct their concerns, which included the name of one of the authors' institutions. The objective of the study was explained to participants, who have been informed at the consent stage of this process that the authors intended to study public opinion on climate change. They were also informed of the length of the survey. The informed consent statement is available in the survey instrument in the Online Annex.

Deception: Information contained in the introductory stages of the survey was factual, including the benchmarking information provided. The conjoint experiment tasks were introduced in hypothetical terms, as policies the government *could* adopt, and respondents were explicitly informed that the countries/policy characteristics displayed were hypothetical, and not factual. As such, the survey did not involve deception. See the survey instrument in the Online Annex for the complete wording choices made by the authors to avoid deception.

Harm and Trauma: we reflected upon the consequences of our survey on participants. The topic studied was not a sensitive one and we anticipated no risk of traumatizing or harming respondents.

Compensation: respondents were drawn from a Qualtrics panel. According to information provided by Qualtrics "[r]espondents will receive an incentive based on the length of the survey, their specific panelist profile, and target acquisition difficulty, amongst other factors. The specific type of rewards varies and may include cash, airline miles, gift cards, redeemable points, charitable donations, sweepstakes entrance, and vouchers."[1] (Qualtrics XM - ESOMAR 28 2019 p.4-5) 28 Questions to Help Buyers of Online Samples, p.4-5).

[1] Qualtrics XM - ESOMAR 28, 2019, *28 Questions to Help Buyers of Online Samples*, last updated April 30, 2019. Obtained through Qualtrics upon authors' request.

2. Survey Instrument

[Welcome and Consent Questions]

Welcome to this survey. We are very grateful for your participation. This survey is carried out for a university-based research project. Its objective is to better understand public opinion concerning policies on climate change. It is solely for scientific purposes, such as publishing academic papers and replicating the findings, and has no commercial or government-related purpose. The survey is anonymous. The information you provide will not be stored or used in any way that could reveal your personal identity. If you have any questions about this research, please contact us at *[email address]*. Our research will only produce meaningful results if you read and think about each question carefully and express your true personal opinion. It will take you no longer than 15 minutes to complete the survey.

If you want to participate in this survey, please select "I have read this statement and agree to participate in this survey" at the bottom of this page. If you do not want to participate, please select "Cancel".

- I have read this statement and agree to participate in this survey
- Cancel

[page break]

[Quota Questions]

Please indicate your gender.

- Female
- Male
- Non-binary

[page break]

Please indicate your age.

- 18-24 years old
- 25-34 years old
- 35-44 years old
- 45-54 years old
- 55-64 years old
- 65-74 years old
- 75-84 years old
- 85 years old or older

[page break]

[Introduction and Attention Check]

We would like to ask some questions about policies on climate change. Scientists attribute climate change mainly to the burning of fossil fuels, which create emissions of carbon

dioxide and other greenhouse gases. From a historical context, developed countries have contributed most to the accumulation of emissions. Because Switzerland is also a developed country, it bears substantial responsibility for climate change. Switzerland is a party to the Paris Agreement of the United Nations convention on climate change. It has adopted policies and possesses technical skills to reduce carbon emissions.

The negative impacts of climate change are visible worldwide in terms of increased frequency and severity of weather events such as floods, heat waves, droughts, storms, etc. While Switzerland also experiences many of these weather events, their impact is more significant in developing countries. This is because developing countries often do not have the resources to adapt. Hence, some people suggest that developed economies such as Switzerland should provide adaptation aid to these countries. In addition, in some cases, damage associated with extreme weather may force people in developing nations to relocate to other countries. According to some, developed countries should accept them as climate migrants.

[page break]

Please indicate which international environmental issue you have just read about.

- Climate Change
- Marine Pollution
- Biodiversity Loss

[answer options, randomized order]

[page break]

[If incorrect answer is selected] The correct answer is “climate change”.

[page break]

[Conjoint Experiment]

In response to the damages caused by climate change induced extreme weather events, the Swiss government could adopt a new policy to support developing countries. The policy could be designed to help countries in different ways. In the next pages, we will present tables, each displaying two possible policies that we ask you to compare. Each option will also include hypothetical characteristics of developing countries, to which the policy would apply. In total, we will show you six tables with such policy comparison. For each pair, compare the two options, and tell us which one you recommend the government of Switzerland to adopt.

Please read very carefully. Some suggestions may seem very similar, but are nevertheless different.

[page break]

[Respondents are randomly allocated to one of the three reference conditions; depending on their group allocation, they are shown the following information before every conjoint task:]

A) OECD Condition:

For reference, in 2019, developed countries members of the Organization for Economic Co-Operation and Development (OECD)…

- have provided on average around 880 million CHF each in bilateral aid focused on climate change,
- have accepted on average 8'300 refugees each.

Please carefully compare these two policies.

For further instructions on the comparison of the tables, please click here.

B) Switzerland Condition:

For reference, in 2019, Switzerland…

- has provided around 380 million CHF in bilateral aid focused on climate change,
- has accepted around 5'500 refugees overall.

Please carefully compare these two policies.

For further instructions on the comparison of the tables, please click here.

C) Control Condition:

Please carefully compare these two policies.

For further instructions on the comparison of the tables, please click here.

[Conjoint table: 6 choice tasks, level of attributes varies randomly]

	**Policy 1**	**Policy 2**
Recipient developing country:	Bangladesh/Philippines/Algeria/Kenya	Bangladesh/Philippines/Algeria/Kenya
Number of climate migrants to accept from	0/250/500/750/1,000/1,250	0/250/500/750/1,000/1,250

this country per year:		
Climate aid to give to this country (CHF) per year:	0/ 30 million/60 million/90 million/120 million	0/ 30 million/60 million/90 million/120 million
Value of Swiss trade with this country:	0/ 500 million /1000 million	0/ 500 million /1000 million
Extreme weather event:	Drought/Sea level rise/ Floods/Cyclones/	Drought/Sea level rise/ Floods/Cyclones/
Percentage of this country's votes in line with Switzerland's position at the UN Security Council:	0 %/ 40 %/ 80 %	0 %/ 40 %/ 80 %

[Below each table, respondents are asked the following choice-question]

Which policy do you support more?

- Policy 1
- Policy 2

[If respondent clicks on 'here' they will again be shown the following instructions:]

We present tables, each displaying two possible policies that we ask you to compare. Each option also includes hypothetical characteristics of developing countries, to which the policy would apply. In total, we will show you six tables with such policy comparison. For each pair, compare the two options, and tell us which one you recommend the government of Switzerland to adopt. Please read very carefully. Some suggestions may seem very similar, but are nevertheless different.

[page break]

[Mechanism Questions]

Please indicate to what extent you agree or disagree with the following statements.

"Immigrants from countries whose languages are similar to those spoken in Switzerland will integrate more easily in Switzerland"

"Immigrants from countries that are culturally similar to Switzerland will integrate more easily in Switzerland"

"I am proud of Swiss culture and history"

"I believe that development aid reduces poverty"

[answer options for each statement: strongly agree; disagree; neither agree, nor agree; agree; strongly agree]

[Socio-demographic Questions]

In which Swiss canton do you live today?

[answer options: select canton from drop-down menu]

In which Swiss municipality do you live today?

[answer options: text, assisted compilation]

[page break]

What is the highest level of education you have attained with a certificate or degree?

- Did not complete compulsory education
- Compulsory education (primary school and middle school)
- Vocational education or training (upper secondary professional education)
- High school or equivalent (upper general secondary education)
- Professional Education Institutions (PEI)
- University or University of Applied Sciences (tertiary education) or higher

[page break]

What is your current occupation?

- Employed
- Self-employed
- Housewife/househusband
- Student
- Retired
- Unemployed
- Prefer not to answer

[page break]

[If employed or self-employed was selected, then:]

In which sector do you currently work?

- Primary sector (e.g. agriculture, sylviculture, mining, etc.)
- Secondary sector (e.g. industry, manufacturing, handicraft, construction, etc.)
- Tertiary sector (e.g. banking, insurance, trading, health, education, public administration, energy/water/waste management, hospitality, transportation, arts and entertainment, scientific and technical professions, etc.)

[page break]

What is your household's net annual income? (After tax and compulsory deductions)

- Less than CHF 23'000
- CHF 23'000 up to less than CHF 62'000
- CHF 62'000 up to less than CHF 92'000
- CHF 92'000 up to less than CHF 123'000
- CHF 123'000 up to less than CHF 154'000
- CHF 154'000 up to less than CHF 231'000
- More than 231'000
- Prefer not to answer

[page break]

Do you have Swiss citizenship?

- Yes
- No
- Prefer not to answer

[page break]

[Political Ideology]

Left, center and right are three concepts often used to characterize political positions. Where do you personally place yourself on the Left-Center-Right spectrum, where 1 means Left and 11 means Right?

[answer: slider 1-11]

[page break]

[Feedback]

Feel free to write about your opinion on this survey, if you wish.
