# Supplementary material for: International climate adaptation assistance: Assessing public support in Switzerland
Source: PLoS One. 2025 Feb 12;20(2):e0317344. doi: 10.1371/journal.pone.0317344 (PMC11819516; doi:10.1371/journal.pone.0317344)
Supplement: S3 Table — (PDF) [file pone.0317344.s003.pdf]

S3 Table. Baseline estimates.

| | MM | | AMCE | |
|---|---|---|---|---|
| Attribute levels | Estimate | p-value | Estimate | p-value |
| **Recipient developing country** | | | | |
| Bangladesh | 0.5192 | 0.0678 | baseline | |
| Algeria | 0.4484 | 0 | -0.0721 | 0.0001 |
| Philippines | 0.5333 | 0.0014 | 0.015 | 0.3679 |
| Kenya | 0.4993 | 0.9412 | -0.0201 | 0.2184 |
| **Number of climate migrants to accept from this country per year** | | | | |
| 0 | 0.5288 | 0.0185 | baseline | |
| 250 | 0.5197 | 0.0768 | -0.0078 | 0.6601 |
| 500 | 0.5135 | 0.1915 | -0.0152 | 0.3671 |
| 750 | 0.5188 | 0.0902 | -0.0079 | 0.6585 |
| 1,000 | 0.4573 | 0.0001 | -0.0706 | 0.0001 |
| 1,250 | 0.4635 | 0.0022 | -0.0645 | 0.0007 |
| **Climate aid to give to this country (CHF) per year** | | | | |
| 0 million | 0.4933 | 0.5298 | baseline | |
| 30 million | 0.5103 | 0.2899 | 0.0202 | 0.1909 |
| 60 million | 0.5 | 1 | 0.0092 | 0.5805 |
| 90 million | 0.5047 | 0.6247 | 0.0131 | 0.4123 |
| 120 million | 0.4916 | 0.3972 | -0.0005 | 0.9783 |
| **Value of Swiss trade with this country** | | | | |
| 0 million | 0.4505 | 0 | baseline | |
| 500 million | 0.5178 | 0.0113 | 0.0695 | 0 |
| 1,000 million | 0.5331 | 0 | 0.0846 | 0 |
| **Extreme weather event** | | | | |
| Drought | 0.5094 | 0.2822 | baseline | |
| Sea level rise | 0.5089 | 0.3055 | -0.0025 | 0.8605 |
| Floods | 0.4912 | 0.3045 | -0.0165 | 0.2396 |
| Cyclones | 0.4903 | 0.2558 | -0.0209 | 0.1321 |
| **UN Security Council votes in line with Switzerland** | | | | |
| 0% | 0.4616 | 0 | baseline | |
| 40% | 0.504 | 0.5642 | 0.0445 | 0.0002 |
| 80% | 0.5363 | 0 | 0.0746 | 0 |
| Number of observations | | | 10568 | |
| R2 | | | 0.0171 | |
| Adj.R2 | | | 0.0153 | |

*Note:* Standard errors for the computation of p-values were clustered by respondent id. For MM estimates that are derived from AMCEs (for details, see Leeper et al., 2018), p-values are computed under null hypothesis that the estimate is equal to 0.5 for the binary choice outcome where respondents choose the preferred policy package when presented with two policy pairs with randomized attribute levels. The label for the attribute level 'Percentage of this country's votes in line with Switzerland's position at the UN Security Council' was replaced with to 'UN Security Council votes in line with Switzerland' for better readability.
