# Supplementary material for: International climate adaptation assistance: Assessing public support in Switzerland
Source: PLoS One. 2025 Feb 12;20(2):e0317344. doi: 10.1371/journal.pone.0317344 (PMC11819516; doi:10.1371/journal.pone.0317344)
Supplement: S5 Table — (PDF) [file pone.0317344.s005.pdf]

S5 Table. Interaction with age.

		MM		AMCE	
Attribute levels	Age	Estimate	p-value	Estimate	p-value
**Recipient developing country**					
Bangladesh	18-34	0.4783	0.2424	baseline	
Algeria	18-34	0.4835	0.3763	0.0027	0.9309
Philippines	18-34	0.5086	0.6408	0.0287	0.335
Kenya	18-34	0.5296	0.1143	0.048	0.101
**Number of climate migrants to accept from this country per year**					
0	18-34	0.4949	0.8247	baseline	
250	18-34	0.5009	0.9644	0.008	0.8067
500	18-34	0.4809	0.3261	-0.0164	0.6023
750	18-34	0.5088	0.6559	0.0115	0.7263
1,000	18-34	0.5051	0.8208	0.0042	0.9049
1,250	18-34	0.5089	0.6902	0.0094	0.7902
**Climate aid to give to this country (CHF) per year**					
0 million	18-34	0.4732	0.1729	baseline	
30 million	18-34	0.4815	0.2614	0.0084	0.758
60 million	18-34	0.5138	0.4619	0.0357	0.255
90 million	18-34	0.5293	0.1094	0.0557	0.0732
120 million	18-34	0.5033	0.8626	0.0277	0.3843
**Value of Swiss trade with this country**					
0 million	18-34	0.4202	0	baseline	
500 million	18-34	0.5172	0.2018	0.0956	0
1,000 million	18-34	0.5639	0	0.1439	0
**Extreme weather event**					
Drought	18-34	0.5061	0.7104	baseline	
Sea level rise	18-34	0.5063	0.695	-0.001	0.9704
Floods	18-34	0.5027	0.8699	0.0055	0.8433
Cyclones	18-34	0.485	0.3287	-0.0182	0.4605
**UN Security Council votes in line with Switzerland**					
0%	18-34	0.4653	0.0139	baseline	
40%	18-34	0.51	0.4692	0.0421	0.0758
80%	18-34	0.526	0.0754	0.0636	0.011
**Recipient developing country**					
Bangladesh	35-64	0.5213	0.1257	baseline	
Algeria	35-64	0.4489	0.0009	-0.071	0.0043
Philippines	35-64	0.5422	0.0032	0.0234	0.2985
Kenya	35-64	0.4879	0.3885	-0.0324	0.1387
**Number of climate migrants to accept from this country per year**					
0	35-64	0.5387	0.0199	baseline	
250	35-64	0.525	0.1056	-0.0148	0.5446
500	35-64	0.5215	0.1305	-0.0166	0.4718
750	35-64	0.5206	0.1731	-0.0126	0.6052
1,000	35-64	0.4569	0.0043	-0.0799	0.0014
1,250	35-64	0.4415	0.0002	-0.0946	0.0002
**Climate aid to give to this country (CHF) per year**					
0 million	35-64	0.5093	0.5223	baseline	
30 million	35-64	0.5194	0.1755	0.0133	0.5428
60 million	35-64	0.5013	0.9205	-0.0032	0.8856
90 million	35-64	0.4928	0.5843	-0.0146	0.4932
120 million	35-64	0.4773	0.103	-0.0306	0.1738

**Value of Swiss trade with this country**					
0 million	35-64	0.454	0	baseline	
500 million	35-64	0.5235	0.0152	0.0722	0
1,000 million	35-64	0.5239	0.0158	0.0718	0
**Extreme weather event**					
Drought	35-64	0.51	0.4106	baseline	
Sea level rise	35-64	0.5111	0.3573	-0.003	0.8773
Floods	35-64	0.4768	0.0441	-0.0337	0.0791
Cyclones	35-64	0.5022	0.8524	-0.0108	0.5762
**UN Security Council votes in line with Switzerland**					
0%	35-64	0.4598	0	baseline	
40%	35-64	0.5036	0.6842	0.0457	0.0045
80%	35-64	0.5382	0.0001	0.0759	0
**Recipient developing country**					
Bangladesh	>65	0.5739	0.0072	baseline	
Algeria	>65	0.3908	0	-0.1893	0
Philippines	>65	0.5477	0.0746	-0.017	0.686
Kenya	>65	0.4857	0.5277	-0.0857	0.0351
**Number of climate migrants to accept from this country per year**					
0	>65	0.5525	0.0642	baseline	
250	>65	0.5355	0.1669	-0.0075	0.8566
500	>65	0.5391	0.0999	-0.0125	0.7553
750	>65	0.5298	0.2892	-0.0251	0.5683
1,000	>65	0.3902	0	-0.1637	0
1,250	>65	0.4563	0.1376	-0.0992	0.0302
**Climate aid to give to this country (CHF) per year**					
0 million	>65	0.4817	0.4737	baseline	
30 million	>65	0.5278	0.1945	0.0618	0.0851
60 million	>65	0.4756	0.2977	-0.0037	0.9273
90 million	>65	0.5	1	0.0214	0.58
120 million	>65	0.5138	0.5205	0.0304	0.4217
**Value of Swiss trade with this country**					
0 million	>65	0.4872	0.4428	baseline	
500 million	>65	0.5016	0.9176	0.0154	0.5596
1,000 million	>65	0.5111	0.5087	0.0287	0.3292
**Extreme weather event**					
Drought	>65	0.5125	0.5119	baseline	
Sea level rise	>65	0.5068	0.7309	-0.0034	0.9165
Floods	>65	0.5156	0.432	0.004	0.8906
Cyclones	>65	0.4653	0.0949	-0.0526	0.1174
**UN Security Council votes in line with Switzerland**					
0%	>65	0.4608	0.0091	baseline	
40%	>65	0.4951	0.7677	0.0393	0.1368
80%	>65	0.547	0.0038	0.0866	0.0015
Number of observations				10568	
R2				0.025	
Adj.R2				0.0199	

Note: Standard errors for the computation of p-values were clustered by respondent id. For MM estimates that are derived from AMCEs (for details, see Leeper et al., 2018), p-values are computed under null hypothesis that the estimate is equal to 0.5 for the binary choice outcome where respondents choose the preferred policy package when presented with two policy pairs with randomized attribute levels. The label for the attribute level 'Percentage of this country's votes in line with Switzerland's position at the UN Security Council' was replaced with to 'UN Security Council votes in line with Switzerland' for better readability.
