# Supplementary material for: International climate adaptation assistance: Assessing public support in Switzerland
Source: PLoS One. 2025 Feb 12;20(2):e0317344. doi: 10.1371/journal.pone.0317344 (PMC11819516; doi:10.1371/journal.pone.0317344)
Supplement: S7 Table — (PDF) [file pone.0317344.s007.pdf]

S7 Table. Interaction with education.

Attribute levels	Education	MM		AMCE	
		Estimate	p-value	Estimate	p-value
**Recipient developing country**					
Bangladesh	University	0.5026	0.8619	baseline	
Algeria	University	0.4871	0.438	-0.0196	0.4693
Philippines	University	0.5183	0.2342	0.0156	0.5144
Kenya	University	0.4929	0.6511	-0.0102	0.6793
**Number of climate migrants to accept from this country per year**					
0	University	0.5	1	baseline	
250	University	0.5014	0.9369	0.0045	0.8647
500	University	0.5085	0.575	0.0112	0.652
750	University	0.5352	0.0351	0.0398	0.1347
1,000	University	0.4639	0.0334	-0.0313	0.2452
1,250	University	0.4924	0.678	-0.0047	0.8693
**Climate aid to give to this country (CHF) per year**					
0 million	University	0.4842	0.336	baseline	
30 million	University	0.5	1	0.0168	0.4858
60 million	University	0.511	0.457	0.0291	0.2461
90 million	University	0.5058	0.7036	0.0184	0.4606
120 million	University	0.4989	0.9419	0.0162	0.5297
**Value of Swiss trade with this country**					
0 million	University	0.4471	0	baseline	
500 million	University	0.5126	0.2478	0.0668	0.0004
1,000 million	University	0.5439	0.0001	0.0973	0
**Extreme weather event**					
Drought	University	0.4972	0.8373	baseline	
Sea level rise	University	0.4951	0.7147	-0.0023	0.9163
Floods	University	0.4995	0.9737	0.0031	0.8886
Cyclones	University	0.5079	0.5409	0.0097	0.6525
**UN Security Council votes in line with Switzerland**					
0%	University	0.4527	0	baseline	
40%	University	0.5027	0.789	0.0526	0.0032
80%	University	0.5455	0	0.0905	0
**Recipient developing country**					
Bangladesh	Professional Education	0.5036	0.8942	baseline	
Algeria	Professional Education	0.4279	0.0077	-0.0806	0.0726
Philippines	Professional Education	0.5561	0.0473	0.0451	0.3063
Kenya	Professional Education	0.5164	0.5143	0.0016	0.9686
**Number of climate migrants to accept from this country per year**					
0	Professional Education	0.5125	0.7081	baseline	
250	Professional Education	0.5158	0.5941	-0.0015	0.9751
500	Professional Education	0.4791	0.4223	-0.0406	0.3669
750	Professional Education	0.5286	0.3341	0.0081	0.8699
1,000	Professional Education	0.4857	0.6033	-0.0364	0.4775
1,250	Professional Education	0.4775	0.4555	-0.0413	0.3968
**Climate aid to give to this country (CHF) per year**					
0 million	Professional Education	0.4874	0.6299	baseline	
30 million	Professional Education	0.5199	0.4127	0.034	0.3694
60 million	Professional Education	0.4805	0.4288	-0.0023	0.9532
90 million	Professional Education	0.5096	0.6715	0.0281	0.4651
120 million	Professional Education	0.5	1	0.0161	0.6815

**Value of Swiss trade with this country**					
0 million	Professional Education	0.4029	0	baseline	
500 million	Professional Education	0.5419	0.0135	0.1345	0
1,000 million	Professional Education	0.5546	0.002	0.1531	0
**Extreme weather event**					
Drought	Professional Education	0.5131	0.5569	baseline	
Sea level rise	Professional Education	0.5012	0.9567	-0.021	0.5768
Floods	Professional Education	0.5084	0.6953	-0.0034	0.9233
Cyclones	Professional Education	0.477	0.2766	-0.0396	0.2377
**UN Security Council votes in line with Switzerland**					
0%	Professional Education	0.4832	0.3817	baseline	
40%	Professional Education	0.4972	0.8773	0.0144	0.6572
80%	Professional Education	0.5191	0.2683	0.0321	0.3136
**Recipient developing country**					
Bangladesh	Vocational	0.5565	0.008	baseline	
Algeria	Vocational	0.3909	0	-0.1647	0
Philippines	Vocational	0.5456	0.0295	-0.0055	0.8685
Kenya	Vocational	0.5007	0.9701	-0.0441	0.1615
**Number of climate migrants to accept from this country per year**					
0	Vocational	0.5892	0.0002	baseline	
250	Vocational	0.5251	0.267	-0.0665	0.0635
500	Vocational	0.546	0.0316	-0.0505	0.1409
750	Vocational	0.4954	0.836	-0.0935	0.0076
1,000	Vocational	0.4422	0.0111	-0.1508	0
1,250	Vocational	0.3953	0	-0.1998	0
**Climate aid to give to this country (CHF) per year**					
0 million	Vocational	0.5093	0.6492	baseline	
30 million	Vocational	0.5302	0.1211	0.0244	0.4008
60 million	Vocational	0.4788	0.2842	-0.0319	0.3301
90 million	Vocational	0.5282	0.1534	0.0173	0.5771
120 million	Vocational	0.4517	0.0176	-0.062	0.0528
**Value of Swiss trade with this country**					
0 million	Vocational	0.4665	0.0204	baseline	
500 million	Vocational	0.5089	0.5227	0.0457	0.0628
1,000 million	Vocational	0.5249	0.0744	0.0606	0.012
**Extreme weather event**					
Drought	Vocational	0.5186	0.2901	baseline	
Sea level rise	Vocational	0.5182	0.3144	-0.0054	0.8463
Floods	Vocational	0.4814	0.2537	-0.0383	0.1602
Cyclones	Vocational	0.4822	0.3251	-0.0402	0.1529
**UN Security Council votes in line with Switzerland**					
0%	Vocational	0.4684	0.017	baseline	
40%	Vocational	0.4967	0.8118	0.0317	0.1686
80%	Vocational	0.5394	0.0083	0.069	0.0041
**Recipient developing country**					
Bangladesh	Other	0.5195	0.4818	baseline	
Algeria	Other	0.4529	0.0619	-0.0639	0.1431
Philippines	Other	0.5299	0.2444	0.0178	0.6811
Kenya	Other	0.4967	0.8956	-0.0215	0.6106
**Number of climate migrants to accept from this country per year**					
0	Other	0.5238	0.4374	baseline	
250	Other	0.558	0.0211	0.0286	0.4837
500	Other	0.5053	0.8398	-0.0159	0.698
750	Other	0.5034	0.8967	-0.0189	0.6637

1,000	Other	0.4377	0.0267	-0.0895	0.0626
1,250	Other	0.476	0.3924	-0.0503	0.2635
**Climate aid to give to this country (CHF) per year**					
0 million	Other	0.4971	0.9185	baseline	
30 million	Other	0.4956	0.8636	0.0051	0.8972
60 million	Other	0.5219	0.3808	0.0223	0.6112
90 million	Other	0.4651	0.1277	-0.0272	0.5138
120 million	Other	0.5212	0.3875	0.0249	0.5561
**Value of Swiss trade with this country**					
0 million	Other	0.4803	0.2846	baseline	
500 million	Other	0.5208	0.2331	0.0409	0.1903
1,000 million	Other	0.4983	0.9246	0.0186	0.5577
**Extreme weather event**					
Drought	Other	0.5212	0.2911	baseline	
Sea level rise	Other	0.5344	0.0716	0.0122	0.716
Floods	Other	0.4678	0.1026	-0.0503	0.1216
Cyclones	Other	0.4679	0.112	-0.0546	0.0955
**UN Security Council votes in line with Switzerland**					
0%	Other	0.4525	0.0068	baseline	
40%	Other	0.5242	0.1721	0.0729	0.0142
80%	Other	0.5256	0.1735	0.0742	0.0163
Number of observations				10568	
R2				0.0256	
Adj.R2				0.0188	

Note: Standard errors for the computation of p-values were clustered by respondent id. For MM estimates that are derived from AMCEs (for details, see Leeper et al., 2018), p-values are computed under null hypothesis that the estimate is equal to 0.5 for the binary choice outcome where respondents choose the preferred policy package when presented with two policy pairs with randomized attribute levels. The label for the attribute level 'Percentage of this country's votes in line with Switzerland's position at the UN Security Council' was replaced with to 'UN Security Council votes in line with Switzerland' for better readability.
