# Supplementary material for: International climate adaptation assistance: Assessing public support in Switzerland
Source: PLoS One. 2025 Feb 12;20(2):e0317344. doi: 10.1371/journal.pone.0317344 (PMC11819516; doi:10.1371/journal.pone.0317344)

S8 Fig. Interaction with income. For more detailed results on point estimates and p-values, see S9 Table.

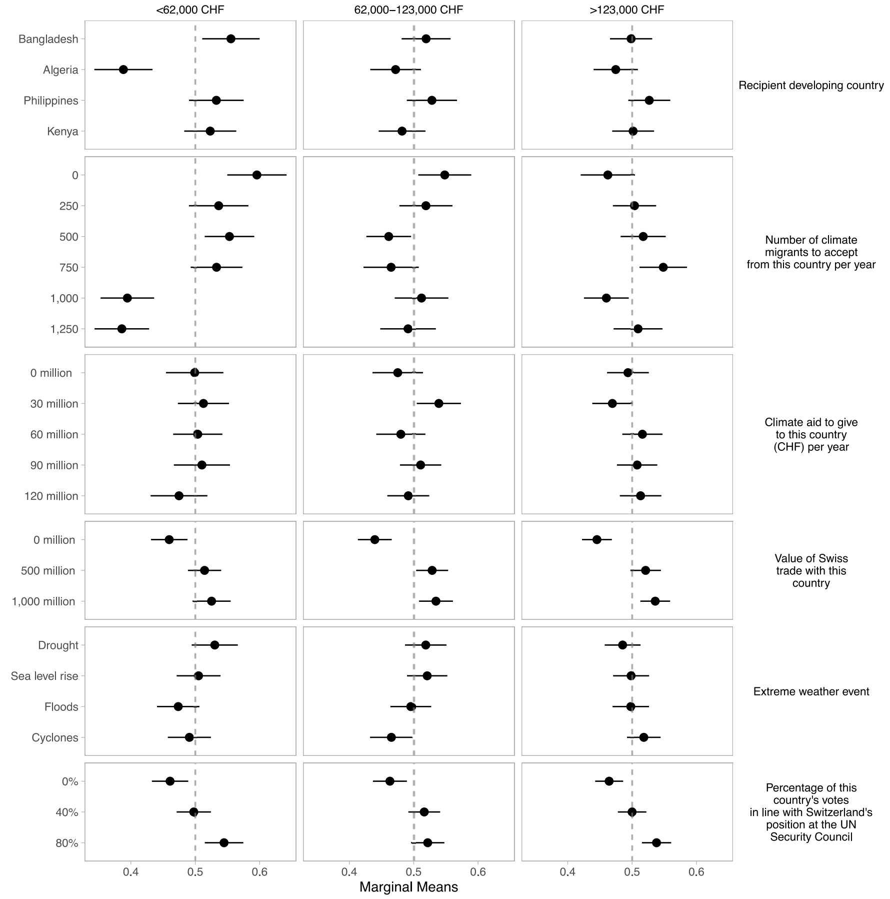

Supplement: S8 Fig — (PDF) [file pone.0317344.s008.pdf]
