# Supplementary material for: International climate adaptation assistance: Assessing public support in Switzerland
Source: PLoS One. 2025 Feb 12;20(2):e0317344. doi: 10.1371/journal.pone.0317344 (PMC11819516; doi:10.1371/journal.pone.0317344)
Supplement: S9 Table — (PDF) [file pone.0317344.s009.pdf]

S9 Table. Interaction with income.

Attribute levels	Income	MM Estimate	MM p-value	AMCE Estimate	AMCE p-value
**Recipient developing country**					
Bangladesh	<62,000 CHF	0.5554	0.015	baseline	
Algeria	<62,000 CHF	0.3884	0	-0.1589	0
Philippines	<62,000 CHF	0.5327	0.1312	-0.0051	0.8818
Kenya	<62,000 CHF	0.5232	0.2603	-0.0168	0.6186
**Number of climate migrants to accept from this country per year**					
0	<62,000 CHF	0.5957	0	baseline	
250	<62,000 CHF	0.5363	0.1231	-0.0527	0.1297
500	<62,000 CHF	0.5531	0.0069	-0.0482	0.1357
750	<62,000 CHF	0.533	0.1071	-0.0648	0.0551
1,000	<62,000 CHF	0.3945	0	-0.1993	0
1,250	<62,000 CHF	0.3859	0	-0.2045	0
**Climate aid to give to this country (CHF) per year**					
0 million	<62,000 CHF	0.499	0.9666	baseline	
30 million	<62,000 CHF	0.5127	0.532	0.0112	0.7198
60 million	<62,000 CHF	0.5039	0.8431	0.005	0.8798
90 million	<62,000 CHF	0.5103	0.6437	0.0075	0.8301
120 million	<62,000 CHF	0.4747	0.2614	-0.0288	0.4278
**Value of Swiss trade with this country**					
0 million	<62,000 CHF	0.4595	0.0052	baseline	
500 million	<62,000 CHF	0.5144	0.274	0.0649	0.0048
1,000 million	<62,000 CHF	0.5253	0.0938	0.0708	0.0056
**Extreme weather event**					
Drought	<62,000 CHF	0.5303	0.0982	baseline	
Sea level rise	<62,000 CHF	0.5051	0.7697	-0.0317	0.2788
Floods	<62,000 CHF	0.4735	0.1152	-0.0596	0.0385
Cyclones	<62,000 CHF	0.4908	0.5908	-0.0536	0.06
**UN Security Council votes in line with Switzerland**					
0%	<62,000 CHF	0.4608	0.0066	baseline	
40%	<62,000 CHF	0.4977	0.8639	0.0367	0.116
80%	<62,000 CHF	0.5447	0.0034	0.0845	0.0011
**Recipient developing country**					
Bangladesh	62,000-123,000 CHF	0.5193	0.3206	baseline	
Algeria	62,000-123,000 CHF	0.4719	0.162	-0.0539	0.1008
Philippines	62,000-123,000 CHF	0.5283	0.1543	0.0049	0.8742
Kenya	62,000-123,000 CHF	0.4819	0.3302	-0.0414	0.1697
**Number of climate migrants to accept from this country per year**					
0	62,000-123,000 CHF	0.5483	0.0216	baseline	
250	62,000-123,000 CHF	0.519	0.3665	-0.0285	0.3732
500	62,000-123,000 CHF	0.4612	0.0288	-0.0857	0.003
750	62,000-123,000 CHF	0.4649	0.1097	-0.0822	0.0103
1,000	62,000-123,000 CHF	0.5122	0.5663	-0.0403	0.2169
1,250	62,000-123,000 CHF	0.4912	0.6907	-0.0588	0.0826
**Climate aid to give to this country (CHF) per year**					
0 million	62,000-123,000 CHF	0.4752	0.2157	baseline	
30 million	62,000-123,000 CHF	0.5391	0.0258	0.0657	0.0185
60 million	62,000-123,000 CHF	0.48	0.3044	0.0021	0.947
90 million	62,000-123,000 CHF	0.5108	0.5107	0.0379	0.2102
120 million	62,000-123,000 CHF	0.4916	0.6149	0.02	0.5013

**Value of Swiss trade with this country**					
0 million	62,000-123,000 CHF	0.4396	0	baseline	
500 million	62,000-123,000 CHF	0.5287	0.0239	0.0875	0.0001
1,000 million	62,000-123,000 CHF	0.5346	0.0103	0.096	0.0001
**Extreme weather event**					
Drought	62,000-123,000 CHF	0.5188	0.2539	baseline	
Sea level rise	62,000-123,000 CHF	0.521	0.1897	0.0011	0.9653
Floods	62,000-123,000 CHF	0.4955	0.7811	-0.0205	0.4425
Cyclones	62,000-123,000 CHF	0.4653	0.0402	-0.057	0.033
**UN Security Council votes in line with Switzerland**					
0%	62,000-123,000 CHF	0.463	0.0061	baseline	
40%	62,000-123,000 CHF	0.5164	0.1943	0.0555	0.0128
80%	62,000-123,000 CHF	0.5218	0.0988	0.055	0.0195
**Recipient developing country**					
Bangladesh	>123,000 CHF	0.4985	0.9271	baseline	
Algeria	>123,000 CHF	0.4746	0.1479	-0.0243	0.4035
Philippines	>123,000 CHF	0.5267	0.1096	0.0304	0.2481
Kenya	>123,000 CHF	0.5016	0.9239	0.0016	0.9535
**Number of climate migrants to accept from this country per year**					
0	>123,000 CHF	0.4623	0.0797	baseline	
250	>123,000 CHF	0.5037	0.8269	0.0412	0.1733
500	>123,000 CHF	0.5172	0.335	0.0521	0.0788
750	>123,000 CHF	0.5484	0.0101	0.0897	0.0044
1,000	>123,000 CHF	0.46	0.0245	-0.0014	0.9638
1,250	>123,000 CHF	0.5092	0.634	0.0454	0.1568
**Climate aid to give to this country (CHF) per year**					
0 million	>123,000 CHF	0.4935	0.6964	baseline	
30 million	>123,000 CHF	0.4694	0.0545	-0.0145	0.564
60 million	>123,000 CHF	0.516	0.3156	0.0321	0.2275
90 million	>123,000 CHF	0.5078	0.6257	0.0173	0.4856
120 million	>123,000 CHF	0.5132	0.423	0.0254	0.3303
**Value of Swiss trade with this country**					
0 million	>123,000 CHF	0.4454	0	baseline	
500 million	>123,000 CHF	0.521	0.0835	0.0771	0.0003
1,000 million	>123,000 CHF	0.5359	0.0024	0.0927	0
**Extreme weather event**					
Drought	>123,000 CHF	0.4853	0.301	baseline	
Sea level rise	>123,000 CHF	0.4985	0.9137	0.0137	0.5636
Floods	>123,000 CHF	0.4979	0.8857	0.0152	0.5191
Cyclones	>123,000 CHF	0.5182	0.1726	0.0355	0.1074
**UN Security Council votes in line with Switzerland**					
0%	>123,000 CHF	0.4643	0.0013	baseline	
40%	>123,000 CHF	0.5	1	0.0375	0.0561
80%	>123,000 CHF	0.538	0.0011	0.0737	0.0001
Number of observations				9536	
R2				0.0277	
Adj.R2				0.022	

Note: Standard errors for the computation of p-values were clustered by respondent id. For MM estimates that are derived from AMCEs (for details, see Leeper et al., 2018), p-values are computed under null hypothesis that the estimate is equal to 0.5 for the binary choice outcome where respondents choose the preferred policy package when presented with two policy pairs with randomized attribute levels. The label for the attribute level 'Percentage of this country's votes in line with Switzerland's position at the UN Security Council' was replaced with to 'UN Security Council votes in line with Switzerland' for better readability.
