# Supplementary material for: International climate adaptation assistance: Assessing public support in Switzerland
Source: PLoS One. 2025 Feb 12;20(2):e0317344. doi: 10.1371/journal.pone.0317344 (PMC11819516; doi:10.1371/journal.pone.0317344)

S10 Fig. Interaction with immigrant language. For more detailed results on point estimates and p-values, see S11 Table.

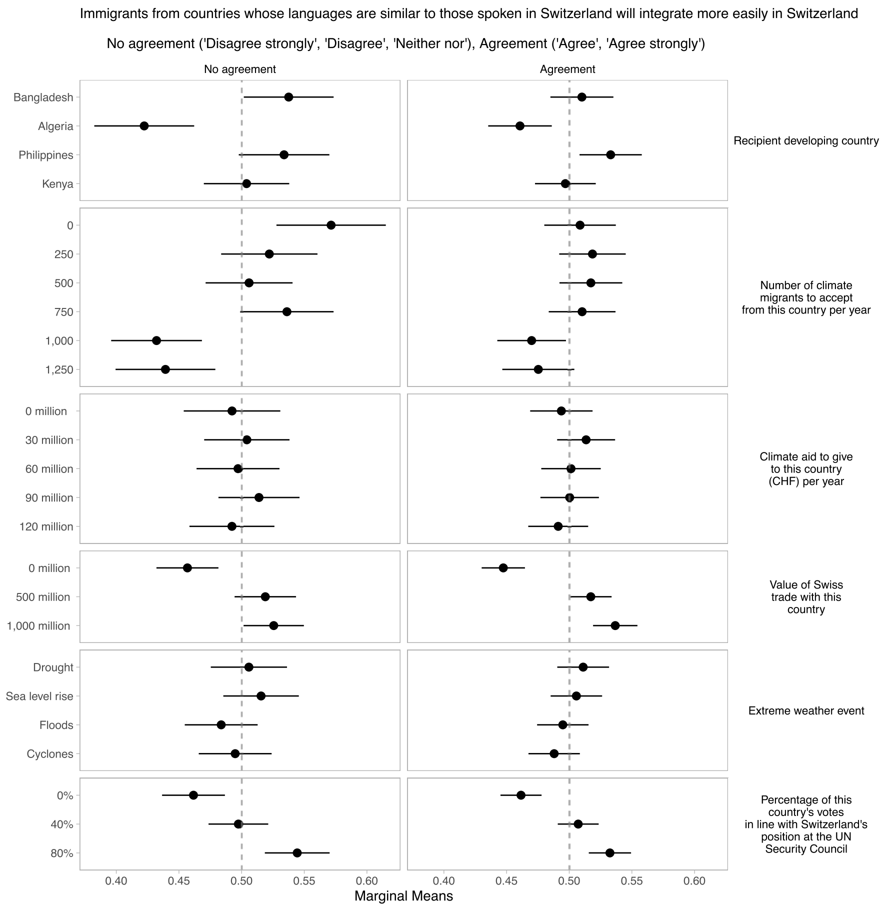

Supplement: S10 Fig — (PDF) [file pone.0317344.s010.pdf]
