# Supplementary material for: International climate adaptation assistance: Assessing public support in Switzerland
Source: PLoS One. 2025 Feb 12;20(2):e0317344. doi: 10.1371/journal.pone.0317344 (PMC11819516; doi:10.1371/journal.pone.0317344)
Supplement: S11 Table — (PDF) [file pone.0317344.s011.pdf]

S11 Table. Interaction with immigrant language.

		MM		AMCE	
Attribute levels	Language	Estimate	p-value	Estimate	p-value
**Recipient developing country**					
Bangladesh	No agreement	0.5376	0.0398	baseline	
Algeria	No agreement	0.4223	0.0001	-0.1147	0.0005
Philippines	No agreement	0.5339	0.0662	-0.0009	0.9744
Kenya	No agreement	0.5039	0.8212	-0.0325	0.2425
**Number of climate migrants to accept from this country per year**					
0	No agreement	0.5714	0.0013	baseline	
250	No agreement	0.5221	0.2595	-0.0507	0.0957
500	No agreement	0.506	0.7362	-0.0679	0.023
750	No agreement	0.5362	0.057	-0.0375	0.2413
1,000	No agreement	0.4321	0.0002	-0.1343	0
1,250	No agreement	0.4392	0.0028	-0.1329	0.0001
**Climate aid to give to this country (CHF) per year**					
0 million	No agreement	0.4924	0.6973	baseline	
30 million	No agreement	0.5042	0.8099	0.0096	0.729
60 million	No agreement	0.4971	0.8652	-0.0011	0.971
90 million	No agreement	0.5139	0.4006	0.0172	0.5543
120 million	No agreement	0.4923	0.6556	-0.0004	0.9891
**Value of Swiss trade with this country**					
0 million	No agreement	0.4568	0.0006	baseline	
500 million	No agreement	0.5189	0.1305	0.0667	0.0019
1,000 million	No agreement	0.5256	0.0368	0.0694	0.0012
**Extreme weather event**					
Drought	No agreement	0.5058	0.7078	baseline	
Sea level rise	No agreement	0.5155	0.3122	0.0007	0.9766
Floods	No agreement	0.4837	0.2726	-0.0227	0.3633
Cyclones	No agreement	0.4949	0.73	-0.0176	0.4763
**UN Security Council votes in line with Switzerland**					
0%	No agreement	0.4616	0.0027	baseline	
40%	No agreement	0.4974	0.8323	0.0415	0.0559
80%	No agreement	0.5444	0.0008	0.084	0.0002
**Recipient developing country**					
Bangladesh	Agreement	0.5101	0.4302	baseline	
Algeria	Agreement	0.4607	0.0024	-0.0521	0.0168
Philippines	Agreement	0.5331	0.0091	0.0231	0.2515
Kenya	Agreement	0.4969	0.8048	-0.0138	0.4959
**Number of climate migrants to accept from this country per year**					
0	Agreement	0.5086	0.5532	baseline	
250	Agreement	0.5185	0.1709	0.0118	0.5855
500	Agreement	0.5173	0.1768	0.0095	0.6433
750	Agreement	0.5103	0.4491	0.0049	0.8207
1,000	Agreement	0.47	0.0319	-0.0401	0.0729
1,250	Agreement	0.4753	0.0927	-0.0323	0.1575
**Climate aid to give to this country (CHF) per year**					
0 million	Agreement	0.4937	0.6212	baseline	
30 million	Agreement	0.5135	0.2535	0.0252	0.1787
60 million	Agreement	0.5014	0.9064	0.0131	0.5198
90 million	Agreement	0.5003	0.9766	0.0099	0.6067
120 million	Agreement	0.4912	0.4708	-0.0009	0.9649

**Value of Swiss trade with this country**					
0 million	Agreement	0.4474	0	baseline	
500 million	Agreement	0.5172	0.0422	0.0711	0
1,000 million	Agreement	0.5367	0	0.0917	0
**Extreme weather event**					
Drought	Agreement	0.5111	0.2914	baseline	
Sea level rise	Agreement	0.5057	0.5857	-0.0034	0.8459
Floods	Agreement	0.4949	0.6253	-0.0127	0.4543
Cyclones	Agreement	0.488	0.2508	-0.0221	0.188
**UN Security Council votes in line with Switzerland**					
0%	Agreement	0.4615	0	baseline	
40%	Agreement	0.5072	0.3887	0.0459	0.0014
80%	Agreement	0.5325	0.0002	0.0695	0
Number of observations				10568	
R2				0.0188	
Adj.R2				0.0153	

Note: Standard errors for the computation of p-values were clustered by respondent id. For MM estimates that are derived from AMCEs (for details, see Leeper et al., 2018), p-values are computed under null hypothesis that the estimate is equal to 0.5 for the binary choice outcome where respondents choose the preferred policy package when presented with two policy pairs with randomized attribute levels. The label for the attribute level 'Percentage of this country's votes in line with Switzerland's position at the UN Security Council' was replaced with to 'UN Security Council votes in line with Switzerland' for better readability.
