# Supplementary material for: International climate adaptation assistance: Assessing public support in Switzerland
Source: PLoS One. 2025 Feb 12;20(2):e0317344. doi: 10.1371/journal.pone.0317344 (PMC11819516; doi:10.1371/journal.pone.0317344)
Supplement: S13 Table — (PDF) [file pone.0317344.s013.pdf]

S13 Table. Interaction with culture.

		MM		AMCE	
Attribute levels	Perceived similarity of immigrants	Estimate	p-value	Estimate	p-value
**Recipient developing country**					
Bangladesh	No agreement	0.5314	0.1626	baseline	
Algeria	No agreement	0.4255	0.0024	-0.1032	0.0101
Philippines	No agreement	0.5178	0.4417	-0.0114	0.7531
Kenya	No agreement	0.5258	0.2306	-0.0019	0.9552
**Number of climate migrants to accept from this country per year**					
0	No agreement	0.5096	0.7289	baseline	
250	No agreement	0.5395	0.0875	0.0332	0.3948
500	No agreement	0.5181	0.3908	0.0104	0.7794
750	No agreement	0.5273	0.2847	0.0188	0.6475
1,000	No agreement	0.4372	0.008	-0.0713	0.0903
1,250	No agreement	0.4712	0.2501	-0.0368	0.3897
**Climate aid to give to this country (CHF) per year**					
0 million	No agreement	0.4932	0.7727	baseline	
30 million	No agreement	0.5246	0.2346	0.0265	0.4403
60 million	No agreement	0.4889	0.5931	-0.0104	0.7742
90 million	No agreement	0.5139	0.4623	0.0219	0.5254
120 million	No agreement	0.4793	0.3261	-0.0128	0.7315
**Value of Swiss trade with this country**					
0 million	No agreement	0.477	0.1675	baseline	
500 million	No agreement	0.5125	0.4301	0.0403	0.1471
1,000 million	No agreement	0.5106	0.498	0.0338	0.2393
**Extreme weather event**					
Drought	No agreement	0.5044	0.8225	baseline	
Sea level rise	No agreement	0.5053	0.7759	-0.0027	0.9329
Floods	No agreement	0.4738	0.1715	-0.034	0.2902
Cyclones	No agreement	0.5158	0.4005	0.0082	0.7882
**UN Security Council votes in line with Switzerland**					
0%	No agreement	0.4906	0.5371	baseline	
40%	No agreement	0.4908	0.5517	0.0032	0.9061
80%	No agreement	0.5199	0.2123	0.0324	0.2195
**Recipient developing country**					
Bangladesh	Agreement	0.5159	0.1796	baseline	
Algeria	Agreement	0.4545	0.0002	-0.0642	0.0017
Philippines	Agreement	0.5373	0.0014	0.0206	0.271
Kenya	Agreement	0.4922	0.4957	-0.024	0.1965
**Number of climate migrants to accept from this country per year**					
0	Agreement	0.534	0.0124	baseline	
250	Agreement	0.5144	0.2562	-0.0182	0.3588
500	Agreement	0.5122	0.3022	-0.0201	0.2884
750	Agreement	0.5167	0.173	-0.0135	0.5001
1,000	Agreement	0.4627	0.0033	-0.0694	0.0007
1,250	Agreement	0.4614	0.0044	-0.0704	0.001
**Climate aid to give to this country (CHF) per year**					
0 million	Agreement	0.4933	0.5771	baseline	
30 million	Agreement	0.5065	0.5545	0.0195	0.2654
60 million	Agreement	0.503	0.7864	0.0135	0.4709
90 million	Agreement	0.5024	0.8311	0.0101	0.5778
120 million	Agreement	0.4947	0.6388	0.0023	0.9023

**Value of Swiss trade with this country**					
0 million	Agreement	0.4436	0	baseline	
500 million	Agreement	0.5191	0.0144	0.0768	0
1,000 million	Agreement	0.5392	0	0.0968	0
**Extreme weather event**					
Drought	Agreement	0.5108	0.2688	baseline	
Sea level rise	Agreement	0.5098	0.3158	-0.0016	0.9207
Floods	Agreement	0.4957	0.6514	-0.0119	0.4462
Cyclones	Agreement	0.4837	0.0895	-0.0283	0.0687
**UN Security Council votes in line with Switzerland**					
0%	Agreement	0.454	0	baseline	
40%	Agreement	0.5076	0.3228	0.0557	0
80%	Agreement	0.5406	0	0.0846	0
Number of observations				10568	
R2				0.0185	
Adj.R2				0.015	

Note: Standard errors for the computation of p-values were clustered by respondent id. For MM estimates that are derived from AMCEs (for details, see Leeper et al., 2018), p-values are computed under null hypothesis that the estimate is equal to 0.5 for the binary choice outcome where respondents choose the preferred policy package when presented with two policy pairs with randomized attribute levels. The label for the attribute level 'Percentage of this country's votes in line with Switzerland's position at the UN Security Council' was replaced with to 'UN Security Council votes in line with Switzerland' for better readability.
