# Supplementary material for: International climate adaptation assistance: Assessing public support in Switzerland
Source: PLoS One. 2025 Feb 12;20(2):e0317344. doi: 10.1371/journal.pone.0317344 (PMC11819516; doi:10.1371/journal.pone.0317344)

S16 Fig. Interaction with the experimental benchmarking. The benchmark concerns other OECD countries (Treatment 1), or Switzerland's existing policies (Treatment 2), compared to no treatment (Control group). For more detailed results on point estimates and p-values, see S17 Table.

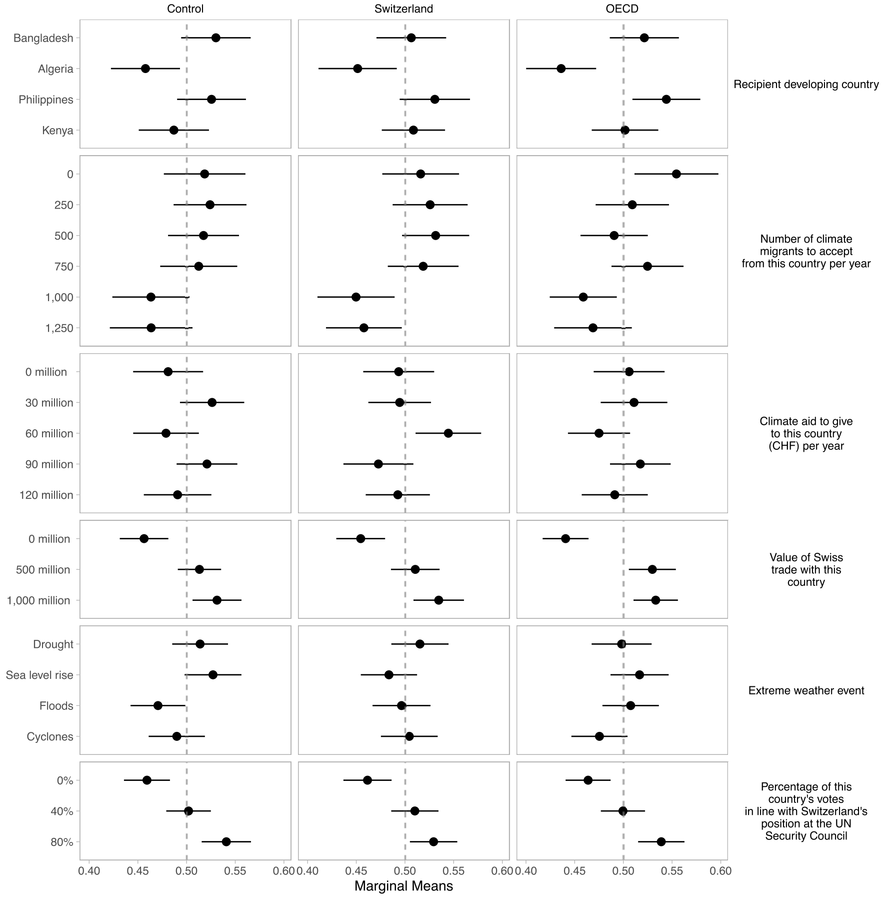

Supplement: S16 Fig — (PDF) [file pone.0317344.s016.pdf]
