# Supplementary material for: International climate adaptation assistance: Assessing public support in Switzerland
Source: PLoS One. 2025 Feb 12;20(2):e0317344. doi: 10.1371/journal.pone.0317344 (PMC11819516; doi:10.1371/journal.pone.0317344)
Supplement: S17 Table — (PDF) [file pone.0317344.s017.pdf]

S17 Table. Interaction with experimental benchmarking.

		MM		AMCE	
Attribute levels	Experimental setup value	Estimate	p-value	Estimate	p-value
**Recipient developing country**					
Bangladesh	ch	0.5065	0.721	baseline	
Algeria	ch	0.4514	0.0178	-0.0582	0.0798
Philippines	ch	0.5307	0.0958	0.0216	0.458
Kenya	ch	0.5087	0.5998	-0.0005	0.9851
**Number of climate migrants to accept from this country per year**					
0	ch	0.5161	0.4226	baseline	
250	ch	0.5259	0.1881	0.0083	0.7902
500	ch	0.5315	0.0733	0.0137	0.6339
750	ch	0.5187	0.3127	0.0001	0.9973
1,000	ch	0.4498	0.013	-0.0648	0.0383
1,250	ch	0.4578	0.0337	-0.055	0.0741
**Climate aid to give to this country (CHF) per year**					
0 million	ch	0.4936	0.7291	baseline	
30 million	ch	0.4946	0.7413	0.005	0.8479
60 million	ch	0.5446	0.0095	0.0523	0.0735
90 million	ch	0.4727	0.1367	-0.0141	0.6231
120 million	ch	0.4927	0.6623	-0.0017	0.9534
**Value of Swiss trade with this country**					
0 million	ch	0.4545	0.0004	baseline	
500 million	ch	0.5106	0.4054	0.0531	0.015
1,000 million	ch	0.5347	0.0088	0.0784	0.0006
**Extreme weather event**					
Drought	ch	0.5154	0.3045	baseline	
Sea level rise	ch	0.4836	0.2644	-0.0314	0.1918
Floods	ch	0.4965	0.8157	-0.0186	0.4514
Cyclones	ch	0.5045	0.7636	-0.0106	0.6602
**UN Security Council votes in line with Switzerland**					
0%	ch	0.4615	0.0023	baseline	
40%	ch	0.5102	0.4122	0.0513	0.0183
80%	ch	0.5294	0.0178	0.0686	0.0016
**Recipient developing country**					
Bangladesh	control	0.5301	0.098	baseline	
Algeria	control	0.4578	0.0197	-0.0722	0.019
Philippines	control	0.5257	0.1549	-0.0008	0.9768
Kenya	control	0.487	0.4789	-0.0398	0.1663
**Number of climate migrants to accept from this country per year**					
0	control	0.5186	0.3847	baseline	
250	control	0.5241	0.2066	0.0075	0.8013
500	control	0.5174	0.3484	0.0006	0.9847
750	control	0.5125	0.5358	-0.0022	0.9461
1,000	control	0.4635	0.0709	-0.0539	0.1043
1,250	control	0.4637	0.0935	-0.0511	0.1376
**Climate aid to give to this country (CHF) per year**					
0 million	control	0.4811	0.3018	baseline	
30 million	control	0.5262	0.1191	0.0455	0.0841
60 million	control	0.4789	0.2207	-0.0008	0.9761
90 million	control	0.521	0.1873	0.038	0.1674
120 million	control	0.4908	0.6035	0.0083	0.7767

**Value of Swiss trade with this country**					
0 million	control	0.4564	0.0006	baseline	
500 million	control	0.5133	0.2424	0.0596	0.0041
1,000 million	control	0.5313	0.0147	0.0749	0.0011
**Extreme weather event**					
Drought	control	0.5139	0.3409	baseline	
Sea level rise	control	0.5272	0.0682	0.0087	0.7179
Floods	control	0.4707	0.0411	-0.0389	0.0953
Cyclones	control	0.49	0.4964	-0.0277	0.2442
**UN Security Council votes in line with Switzerland**					
0%	control	0.4593	0.0007	baseline	
40%	control	0.5021	0.8596	0.0431	0.0326
80%	control	0.5409	0.0016	0.0798	0.0002
**Recipient developing country**					
Bangladesh	oecd	0.5215	0.2342	baseline	
Algeria	oecd	0.4361	0.0005	-0.085	0.005
Philippines	oecd	0.5442	0.013	0.0252	0.3687
Kenya	oecd	0.5017	0.9227	-0.0203	0.4823
**Number of climate migrants to accept from this country per year**					
0	oecd	0.5545	0.0132	baseline	
250	oecd	0.5092	0.6327	-0.0403	0.1936
500	oecd	0.4905	0.5913	-0.0618	0.0393
750	oecd	0.5248	0.1891	-0.0239	0.4448
1,000	oecd	0.4588	0.0191	-0.0932	0.0023
1,250	oecd	0.4688	0.1253	-0.0848	0.0121
**Climate aid to give to this country (CHF) per year**					
0 million	oecd	0.5059	0.7501	baseline	
30 million	oecd	0.5109	0.5305	0.0098	0.7289
60 million	oecd	0.475	0.1246	-0.0257	0.3697
90 million	oecd	0.5174	0.2728	0.0128	0.6368
120 million	oecd	0.4912	0.6091	-0.0093	0.7468
**Value of Swiss trade with this country**					
0 million	oecd	0.4407	0	baseline	
500 million	oecd	0.5298	0.0156	0.0953	0
1,000 million	oecd	0.5332	0.0043	0.098	0
**Extreme weather event**					
Drought	oecd	0.4982	0.9097	baseline	
Sea level rise	oecd	0.5166	0.2745	0.0176	0.4962
Floods	oecd	0.5075	0.6128	0.0116	0.6392
Cyclones	oecd	0.4754	0.0952	-0.0234	0.3386
**UN Security Council votes in line with Switzerland**					
0%	oecd	0.4638	0.0021	baseline	
40%	oecd	0.4996	0.9699	0.0391	0.0541
80%	oecd	0.5389	0.0014	0.0762	0.0003
Number of observations				10568	
R2				0.02	
Adj.R2				0.0149	

Note: Standard errors for the computation of p-values were clustered by respondent id. For MM estimates that are derived from AMCEs (for details, see Leeper et al., 2018), p-values are computed under null hypothesis that the estimate is equal to 0.5 for the binary choice outcome where respondents choose the preferred policy package when presented with two policy pairs with randomized attribute levels. The label for the attribute level 'Percentage of this country's votes in line with Switzerland's position at the UN Security Council' was replaced with to 'UN Security Council votes in line with Switzerland' for better readability.
