# Supplementary material for: International climate adaptation assistance: Assessing public support in Switzerland
Source: PLoS One. 2025 Feb 12;20(2):e0317344. doi: 10.1371/journal.pone.0317344 (PMC11819516; doi:10.1371/journal.pone.0317344)
Supplement: S18 Table — (PDF) [file pone.0317344.s018.pdf]

S18 Table. Interaction with Swiss trade.

		MM		AMCE	
Attribute levels	Value of Swiss trade	Estimate	p-value	Estimate	p-value
**Recipient developing country**					
Bangladesh	0 million	0.4631	0.0339	baseline	
Algeria	0 million	0.3909	0	-0.0707	0.0099
Philippines	0 million	0.4855	0.4011	0.0228	0.3665
Kenya	0 million	0.4593	0.0206	-0.0025	0.9233
**Number of climate migrants to accept from this country per year**					
0	0 million	0.4567	0.0353	baseline	
250	0 million	0.4818	0.3625	0.031	0.2825
500	0 million	0.4628	0.0609	0.0043	0.8799
750	0 million	0.4618	0.0555	0.0072	0.8119
1,000	0 million	0.4236	0.0001	-0.0308	0.2896
1,250	0 million	0.4148	0	-0.039	0.1978
**Climate aid to give to this country (CHF) per year**					
0 million	0 million	0.4655	0.0753	baseline	
30 million	0 million	0.4554	0.0125	-0.0095	0.7239
60 million	0 million	0.4373	0.0005	-0.0301	0.2597
90 million	0 million	0.45	0.0039	-0.0161	0.5442
120 million	0 million	0.4448	0.0026	-0.0195	0.4717
**Extreme weather event**					
Drought	0 million	0.4261	0	baseline	
Sea level rise	0 million	0.4612	0.0167	0.0325	0.1789
Floods	0 million	0.4548	0.0038	0.0256	0.2822
Cyclones	0 million	0.4591	0.0134	0.0298	0.212
**UN Security Council votes in line with Switzerland**					
0%	0 million	0.4151	0	baseline	
40%	0 million	0.4551	0.0008	0.0431	0.0365
80%	0 million	0.4821	0.1903	0.0677	0.001
**Recipient developing country**					
Bangladesh	500 million	0.5225	0.2075	baseline	
Algeria	500 million	0.4725	0.1206	-0.0486	0.0757
Philippines	500 million	0.5675	0.0001	0.0463	0.071
Kenya	500 million	0.5089	0.5969	-0.0128	0.6181
**Number of climate migrants to accept from this country per year**					
0	500 million	0.5455	0.0364	baseline	
250	500 million	0.5198	0.3198	-0.0251	0.4079
500	500 million	0.5389	0.058	-0.0062	0.8378
750	500 million	0.5556	0.0057	0.0119	0.6873
1,000	500 million	0.4728	0.1754	-0.0736	0.0191
1,250	500 million	0.4812	0.3648	-0.0636	0.0418
**Climate aid to give to this country (CHF) per year**					
0 million	500 million	0.5167	0.37	baseline	
30 million	500 million	0.5279	0.1126	0.0117	0.6563
60 million	500 million	0.5189	0.3091	-0.0008	0.9762
90 million	500 million	0.5202	0.2626	-0.0004	0.9878
120 million	500 million	0.505	0.7835	-0.0135	0.6162
**Extreme weather event**					
Drought	500 million	0.5443	0.0097	baseline	
Sea level rise	500 million	0.518	0.2522	-0.0288	0.2299
Floods	500 million	0.5114	0.4941	-0.0329	0.1749

Cyclones	500 million	0.4983	0.9166	-0.0457	0.0626
**UN Security Council votes in line with Switzerland**					
0%	500 million	0.4789	0.1222	baseline	
40%	500 million	0.518	0.1871	0.0414	0.0423
80%	500 million	0.5591	0	0.0799	0.0001
**Recipient developing country**					
Bangladesh	1,000 million	0.5742	0	baseline	
Algeria	1,000 million	0.4799	0.2435	-0.0965	0.0002
Philippines	1,000 million	0.5491	0.004	-0.0235	0.338
Kenya	1,000 million	0.5306	0.0751	-0.0467	0.0607
**Number of climate migrants to accept from this country per year**					
0	1,000 million	0.5884	0	baseline	
250	1,000 million	0.5586	0.0035	-0.0292	0.3275
500	1,000 million	0.5406	0.0334	-0.0425	0.1284
750	1,000 million	0.5428	0.0367	-0.0439	0.1313
1,000	1,000 million	0.4757	0.2514	-0.1112	0.0002
1,250	1,000 million	0.494	0.7624	-0.0929	0.0015
**Climate aid to give to this country (CHF) per year**					
0 million	1,000 million	0.4986	0.942	baseline	
30 million	1,000 million	0.5475	0.0091	0.0554	0.0327
60 million	1,000 million	0.5471	0.0161	0.058	0.0396
90 million	1,000 million	0.5482	0.0104	0.0554	0.0448
120 million	1,000 million	0.5263	0.1562	0.0296	0.2826
**Extreme weather event**					
Drought	1,000 million	0.5578	0.0005	baseline	
Sea level rise	1,000 million	0.5481	0.003	-0.0147	0.5406
Floods	1,000 million	0.5119	0.4842	-0.0447	0.0681
Cyclones	1,000 million	0.5127	0.4606	-0.0458	0.0639
**UN Security Council votes in line with Switzerland**					
0%	1,000 million	0.4909	0.4984	baseline	
40%	1,000 million	0.5409	0.0042	0.0509	0.014
80%	1,000 million	0.5698	0	0.0768	0.0002
Number of observations				10568	
R2				0.0197	
Adj.R2				0.0151	

Note: Standard errors for the computation of p-values were clustered by respondent id. For MM estimates that are derived from AMCEs (for details, see Leeper et al., 2018), p-values are computed under null hypothesis that the estimate is equal to 0.5 for the binary choice outcome where respondents choose the preferred policy package when presented with two policy pairs with randomized attribute levels. The label for the attribute level 'Percentage of this country's votes in line with Switzerland's position at the UN Security Council' was replaced with to 'UN Security Council votes in line with Switzerland' for better readability.
