# Supplementary material for: International climate adaptation assistance: Assessing public support in Switzerland
Source: PLoS One. 2025 Feb 12;20(2):e0317344. doi: 10.1371/journal.pone.0317344 (PMC11819516; doi:10.1371/journal.pone.0317344)
Supplement: S19 Table — (PDF) [file pone.0317344.s019.pdf]

S19 Table. Interaction with UN Security Council votes.

Attribute levels	Votes in line with Switzerland	MM		AMCE	
		Estimate	p-value	Estimate	p-value
**Recipient developing country**					
Bangladesh	0%	0.4822	0.2868	baseline	
Algeria	0%	0.4117	0	-0.0721	0.0062
Philippines	0%	0.4938	0.7283	0.0118	0.6453
Kenya	0%	0.458	0.0147	-0.0252	0.3181
**Number of climate migrants to accept from this country per year**					
0	0%	0.4826	0.401	baseline	
250	0%	0.5008	0.9673	0.0233	0.4312
500	0%	0.4635	0.0663	-0.0196	0.4827
750	0%	0.4684	0.1087	-0.016	0.5858
1,000	0%	0.4304	0.0004	-0.0528	0.077
1,250	0%	0.4239	0.0002	-0.0578	0.0561
**Climate aid to give to this country (CHF) per year**					
0 million	0%	0.4755	0.2034	baseline	
30 million	0%	0.4772	0.213	0.0019	0.9419
60 million	0%	0.4583	0.0203	-0.0165	0.5523
90 million	0%	0.4515	0.0057	-0.0242	0.3651
120 million	0%	0.4454	0.0012	-0.0297	0.2585
**Value of Swiss trade with this country**					
0 million	0%	0.4151	0	baseline	
500 million	0%	0.4789	0.1222	0.0673	0.0009
1,000 million	0%	0.4909	0.4984	0.0798	0.0001
**Extreme weather event**					
Drought	0%	0.4642	0.0316	baseline	
Sea level rise	0%	0.47	0.0653	0.0033	0.8916
Floods	0%	0.4641	0.0191	0.0007	0.9761
Cyclones	0%	0.4478	0.0016	-0.0175	0.4723
**Recipient developing country**					
Bangladesh	40%	0.5287	0.1121	baseline	
Algeria	40%	0.4554	0.0083	-0.0725	0.0064
Philippines	40%	0.539	0.0262	0.0135	0.6091
Kenya	40%	0.4957	0.7976	-0.0298	0.2484
**Number of climate migrants to accept from this country per year**					
0	40%	0.5202	0.3636	baseline	
250	40%	0.5096	0.6322	-0.0118	0.6951
500	40%	0.531	0.1122	0.0081	0.7922
750	40%	0.5321	0.1198	0.0165	0.5976
1,000	40%	0.4636	0.0602	-0.0567	0.0637
1,250	40%	0.4732	0.1919	-0.0431	0.1811
**Climate aid to give to this country (CHF) per year**					
0 million	40%	0.4798	0.2776	baseline	
30 million	40%	0.5296	0.1082	0.0513	0.0551
60 million	40%	0.5139	0.4541	0.0379	0.1591
90 million	40%	0.5092	0.6256	0.0306	0.2732
120 million	40%	0.4871	0.4829	0.0087	0.7514
**Value of Swiss trade with this country**					
0 million	40%	0.4551	0.0008	baseline	
500 million	40%	0.518	0.1871	0.0631	0.0018
1,000 million	40%	0.5409	0.0042	0.086	0

**Extreme weather event**					
Drought	40%	0.5267	0.1029	baseline	
Sea level rise	40%	0.5087	0.5709	-0.0197	0.3979
Floods	40%	0.4887	0.5022	-0.0401	0.1026
Cyclones	40%	0.4911	0.5823	-0.0385	0.1018
**Recipient developing country**					
Bangladesh	80%	0.5491	0.0035	baseline	
Algeria	80%	0.4798	0.2482	-0.0746	0.0042
Philippines	80%	0.5686	0.0001	0.0177	0.4791
Kenya	80%	0.5465	0.0066	-0.0051	0.8373
**Number of climate migrants to accept from this country per year**					
0	80%	0.5822	0	baseline	
250	80%	0.5505	0.0123	-0.0358	0.2143
500	80%	0.5478	0.0145	-0.0338	0.236
750	80%	0.5604	0.0029	-0.0242	0.4118
1,000	80%	0.4801	0.3206	-0.104	0.0002
1,250	80%	0.4939	0.7725	-0.0921	0.002
**Climate aid to give to this country (CHF) per year**					
0 million	80%	0.5259	0.1728	baseline	
30 million	80%	0.5267	0.1565	0.0041	0.8797
60 million	80%	0.5294	0.1289	0.0045	0.8714
90 million	80%	0.5543	0.0032	0.0308	0.2604
120 million	80%	0.5442	0.0199	0.0201	0.4735
**Value of Swiss trade with this country**					
0 million	80%	0.4821	0.1903	baseline	
500 million	80%	0.5591	0	0.0806	0.0001
1,000 million	80%	0.5698	0	0.0906	0
**Extreme weather event**					
Drought	80%	0.538	0.0209	baseline	
Sea level rise	80%	0.5482	0.0029	0.009	0.7044
Floods	80%	0.5236	0.166	-0.0104	0.6718
Cyclones	80%	0.5347	0.0367	-0.0094	0.7025
Number of observations				10568	
R2				0.0184	
Adj.R2				0.0141	

Note: Standard errors for the computation of p-values were clustered by respondent id. For MM estimates that are derived from AMCEs (for details, see Leeper et al., 2018), p-values are computed under null hypothesis that the estimate is equal to 0.5 for the binary choice outcome where respondents choose the preferred policy package when presented with two policy pairs with randomized attribute levels. The label for the attribute level 'Percentage of this country's votes in line with Switzerland's position at the UN Security Council' was replaced with to 'UN Security Council votes in line with Switzerland' for better readability.
