# Supplementary material for: International climate adaptation assistance: Assessing public support in Switzerland
Source: PLoS One. 2025 Feb 12;20(2):e0317344. doi: 10.1371/journal.pone.0317344 (PMC11819516; doi:10.1371/journal.pone.0317344)

S20 Fig. Average Marginal Interaction Effect (AMIE). Panel a shows the AMIE for the conjoint attribute *value of Swiss Trade with this country.* Panel b shows the AMIE for the conjoint attribute *percentage of this country's votes in line with Switzerland's position at the UN Security Council'.* The horizontal axis shows the index of the treatment combinations ordered by the treatment effect. The vertical axis shows the AMIE. Colors represent the recipient developing country. For details, see S21 and S22 Tables.

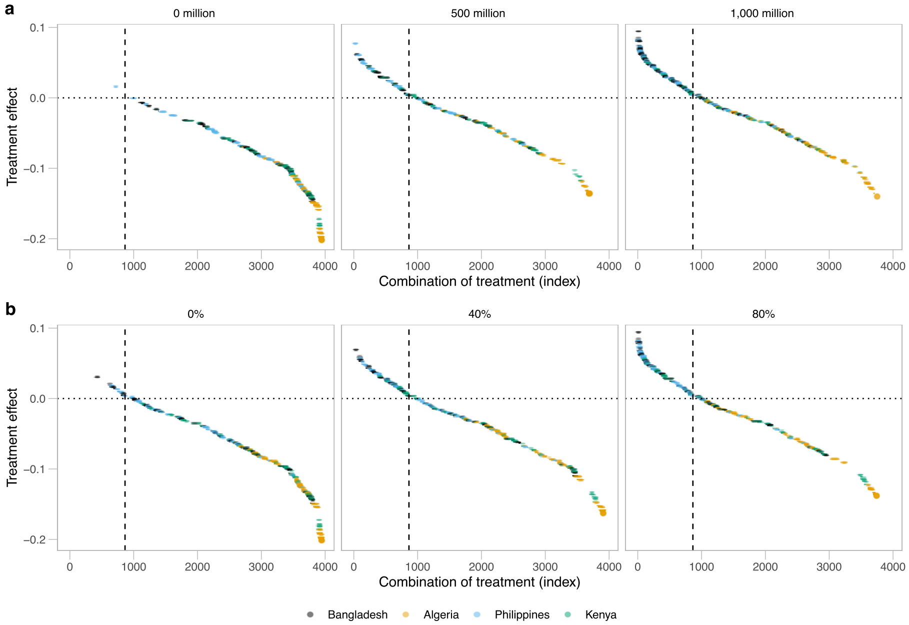

Supplement: S20 Fig — (PDF) [file pone.0317344.s020.pdf]
