# Supplementary material for: International climate adaptation assistance: Assessing public support in Switzerland
Source: PLoS One. 2025 Feb 12;20(2):e0317344. doi: 10.1371/journal.pone.0317344 (PMC11819516; doi:10.1371/journal.pone.0317344)
Supplement: S21 Table — (PDF) [file pone.0317344.s021.pdf]

S21 Table. 50 treatment combinations with the highest treatment effect.

Treatment effect	Recipient developing country	Number of climate migrants	Climate aid	Value of Swiss trade	Extreme weather event	UN Security Council with Switzerland
0.0904460	Bangladesh	0	30 million	1,000 million	Drought	80%
0.0904460	Bangladesh	0	60 million	1,000 million	Drought	80%
0.0904460	Bangladesh	0	120 million	1,000 million	Drought	80%
0.0812589	Bangladesh	0	0 million	1,000 million	Drought	80%
0.0783311	Philippines	250	30 million	1,000 million	Sea level rise	80%
0.0783311	Philippines	250	90 million	1,000 million	Sea level rise	80%
0.0783311	Philippines	250	120 million	1,000 million	Sea level rise	80%
0.0783311	Philippines	250	60 million	1,000 million	Sea level rise	80%
0.0763574	Bangladesh	0	120 million	1,000 million	Floods	80%
0.0763574	Bangladesh	0	30 million	1,000 million	Floods	80%
0.0763574	Bangladesh	0	90 million	1,000 million	Cyclones	80%
0.0763574	Bangladesh	0	90 million	1,000 million	Floods	80%
0.0763574	Bangladesh	0	120 million	1,000 million	Sea level rise	80%
0.0763574	Bangladesh	0	60 million	1,000 million	Floods	80%
0.0763574	Bangladesh	0	60 million	1,000 million	Cyclones	80%
0.0763574	Bangladesh	0	30 million	1,000 million	Cyclones	80%
0.0763574	Bangladesh	0	90 million	1,000 million	Sea level rise	80%
0.0763574	Bangladesh	0	60 million	1,000 million	Sea level rise	80%
0.0763574	Bangladesh	0	30 million	1,000 million	Sea level rise	80%
0.0763574	Bangladesh	0	120 million	1,000 million	Cyclones	80%
0.0734273	Philippines	250	60 million	500 million	Sea level rise	80%
0.0734273	Philippines	250	0 million	500 million	Sea level rise	80%
0.0734273	Philippines	250	90 million	500 million	Sea level rise	80%
0.0734273	Philippines	250	120 million	500 million	Sea level rise	80%
0.0691440	Philippines	250	0 million	1,000 million	Sea level rise	80%
0.0690796	Philippines	0	120 million	1,000 million	Sea level rise	80%
0.0690796	Philippines	0	30 million	1,000 million	Sea level rise	80%
0.0690796	Philippines	0	90 million	1,000 million	Sea level rise	80%
0.0690796	Philippines	0	60 million	1,000 million	Sea level rise	80%
0.0671703	Bangladesh	0	0 million	1,000 million	Cyclones	80%
0.0671703	Bangladesh	0	0 million	1,000 million	Sea level rise	80%
0.0655464	Bangladesh	0	90 million	1,000 million	Drought	40%
0.0655464	Bangladesh	0	30 million	1,000 million	Drought	40%
0.0655464	Bangladesh	0	120 million	1,000 million	Drought	40%
0.0637616	Philippines	0	90 million	1,000 million	Drought	80%
0.0637616	Philippines	0	60 million	1,000 million	Drought	80%

0.0637616	Philippines	0	120 million	1,000 million	Drought	80%
0.0637616	Philippines	0	30 million	1,000 million	Drought	80%
0.0619713	Philippines	750	120 million	1,000 million	Sea level rise	80%
0.0619713	Philippines	500	120 million	1,000 million	Sea level rise	80%
0.0619713	Philippines	750	60 million	1,000 million	Sea level rise	80%
0.0619713	Philippines	500	30 million	1,000 million	Sea level rise	80%
0.0619713	Philippines	750	30 million	1,000 million	Sea level rise	80%
0.0619713	Philippines	750	90 million	1,000 million	Sea level rise	80%
0.0619713	Philippines	500	90 million	1,000 million	Sea level rise	80%
0.0598925	Philippines	0	0 million	1,000 million	Sea level rise	80%
0.0597880	Bangladesh	250	90 million	1,000 million	Sea level rise	80%
0.0597880	Bangladesh	250	120 million	1,000 million	Sea level rise	80%
0.0597880	Bangladesh	250	60 million	1,000 million	Sea level rise	80%
0.0597880	Bangladesh	250	30 million	1,000 million	Sea level rise	80%
