# Supplementary material for: International climate adaptation assistance: Assessing public support in Switzerland
Source: PLoS One. 2025 Feb 12;20(2):e0317344. doi: 10.1371/journal.pone.0317344 (PMC11819516; doi:10.1371/journal.pone.0317344)
Supplement: S22 Table — (PDF) [file pone.0317344.s022.pdf]

S22 Table. 50 treatment combinations with the lowest treatment effect.

Treatment effect	Recipient developing country	Number of climate migrants	Climate aid	Value of Swiss trade	Extreme weather event	UN Security Council with Switzerland
-0.1579033	Algeria	1,000	90 million	0 million	Cyclones	40%
-0.1579033	Algeria	1,000	90 million	0 million	Sea level rise	40%
-0.1579033	Algeria	1,000	0 million	0 million	Cyclones	40%
-0.1579033	Algeria	1,000	60 million	0 million	Floods	40%
-0.1627947	Algeria	1,000	120 million	0 million	Drought	40%
-0.1627947	Algeria	1,000	60 million	0 million	Drought	40%
-0.1627947	Algeria	1,000	0 million	0 million	Drought	40%
-0.1627947	Algeria	1,000	30 million	0 million	Drought	40%
-0.1757793	Kenya	1,250	120 million	0 million	Sea level rise	0%
-0.1757793	Kenya	1,250	120 million	0 million	Cyclones	0%
-0.1823293	Kenya	1,000	120 million	0 million	Sea level rise	0%
-0.1823293	Kenya	1,000	120 million	0 million	Cyclones	0%
-0.1823293	Kenya	1,000	120 million	0 million	Floods	0%
-0.1854448	Kenya	1,000	120 million	0 million	Drought	0%
-0.1899828	Algeria	1,250	120 million	0 million	Sea level rise	0%
-0.1899828	Algeria	1,250	0 million	0 million	Cyclones	0%
-0.1899828	Algeria	1,250	60 million	0 million	Floods	0%
-0.1899828	Algeria	1,250	90 million	0 million	Floods	0%
-0.1899828	Algeria	1,250	120 million	0 million	Cyclones	0%
-0.1899828	Algeria	1,250	30 million	0 million	Sea level rise	0%
-0.1899828	Algeria	1,250	90 million	0 million	Cyclones	0%
-0.1899828	Algeria	1,250	30 million	0 million	Floods	0%
-0.1899828	Algeria	1,250	90 million	0 million	Sea level rise	0%
-0.1899828	Algeria	1,250	0 million	0 million	Sea level rise	0%
-0.1899828	Algeria	1,250	60 million	0 million	Cyclones	0%
-0.1899828	Algeria	1,250	120 million	0 million	Floods	0%
-0.1948742	Algeria	1,250	90 million	0 million	Drought	0%
-0.1948742	Algeria	1,250	0 million	0 million	Drought	0%
-0.1948742	Algeria	1,250	120 million	0 million	Drought	0%
-0.1948742	Algeria	1,250	60 million	0 million	Drought	0%
-0.1948742	Algeria	1,250	30 million	0 million	Drought	0%
-0.1965328	Algeria	1,000	30 million	0 million	Sea level rise	0%
-0.1965328	Algeria	1,000	60 million	0 million	Floods	0%
-0.1965328	Algeria	1,000	0 million	0 million	Floods	0%
-0.1965328	Algeria	1,000	30 million	0 million	Floods	0%
-0.1965328	Algeria	1,000	120 million	0 million	Sea level rise	0%

-0.1965328	Algeria	1,000	120 million	0 million	Floods	0%
-0.1965328	Algeria	1,000	90 million	0 million	Sea level rise	0%
-0.1965328	Algeria	1,000	120 million	0 million	Cyclones	0%
-0.1965328	Algeria	1,000	30 million	0 million	Cyclones	0%
-0.1965328	Algeria	1,000	0 million	0 million	Cyclones	0%
-0.1965328	Algeria	1,000	60 million	0 million	Cyclones	0%
-0.1965328	Algeria	1,000	90 million	0 million	Cyclones	0%
-0.1965328	Algeria	1,000	90 million	0 million	Floods	0%
-0.1965328	Algeria	1,000	60 million	0 million	Sea level rise	0%
-0.2014242	Algeria	1,000	60 million	0 million	Drought	0%
-0.2014242	Algeria	1,000	30 million	0 million	Drought	0%
-0.2014242	Algeria	1,000	0 million	0 million	Drought	0%
-0.2014242	Algeria	1,000	120 million	0 million	Drought	0%
-0.2014242	Algeria	1,000	90 million	0 million	Drought	0%
