# Supplementary material for: International climate adaptation assistance: Assessing public support in Switzerland
Source: PLoS One. 2025 Feb 12;20(2):e0317344. doi: 10.1371/journal.pone.0317344 (PMC11819516; doi:10.1371/journal.pone.0317344)
Supplement: S24 Table — (PDF) [file pone.0317344.s024.pdf]

S24 Table. Interaction with the left-right political spectrum

		MM		AMCE	
Attribute levels	Left-right	Estimate	p-value	Estimate	p-value
**Recipient developing country**					
Bangladesh	Left	0.5314	0.1289	baseline	
Algeria	Left	0.4523	0.0293	-0.0804	0.024
Philippines	Left	0.4906	0.6587	-0.0393	0.2308
Kenya	Left	0.5207	0.2781	-0.0123	0.7061
**Number of climate migrants to accept from this country per year**					
0	Left	0.5291	0.2034	baseline	
250	Left	0.5021	0.9171	-0.0314	0.3161
500	Left	0.5255	0.2377	-0.005	0.8777
750	Left	0.5089	0.6944	-0.0184	0.6063
1,000	Left	0.4815	0.4	-0.0521	0.1237
1,250	Left	0.4516	0.0314	-0.0829	0.0201
**Climate aid to give to this country (CHF) per year**					
0 million	Left	0.4629	0.0917	baseline	
30 million	Left	0.5326	0.086	0.0725	0.0189
60 million	Left	0.4953	0.8092	0.0305	0.3722
90 million	Left	0.5185	0.3357	0.057	0.0793
120 million	Left	0.488	0.5517	0.0271	0.4228
**Value of Swiss trade with this country**					
0 million	Left	0.4453	0.0001	baseline	
500 million	Left	0.5223	0.0881	0.0819	0.0005
1,000 million	Left	0.5347	0.0099	0.0939	0.0001
**Extreme weather event**					
Drought	Left	0.5408	0.0193	baseline	
Sea level rise	Left	0.5213	0.1981	-0.0243	0.3943
Floods	Left	0.4879	0.4578	-0.0528	0.0567
Cyclones	Left	0.4441	0.0014	-0.1053	0.0002
**UN Security Council votes in line with Switzerland**					
0%	Left	0.4502	0.0003	baseline	
40%	Left	0.5147	0.3088	0.0647	0.0075
80%	Left	0.5363	0.0136	0.0845	0.0005
**Recipient developing country**					
Bangladesh	Centrist	0.5402	0.1058	baseline	
Algeria	Centrist	0.4185	0.0005	-0.1282	0.0018
Philippines	Centrist	0.5174	0.4844	-0.0371	0.3462
Kenya	Centrist	0.5261	0.262	-0.0226	0.5408
**Number of climate migrants to accept from this country per year**					
0	Centrist	0.5446	0.1253	baseline	
250	Centrist	0.5398	0.144	0.0019	0.9651
500	Centrist	0.5015	0.9506	-0.042	0.2966
750	Centrist	0.474	0.3273	-0.0702	0.0825
1,000	Centrist	0.4675	0.2049	-0.073	0.0829
1,250	Centrist	0.4749	0.3275	-0.0655	0.1195
**Climate aid to give to this country (CHF) per year**					
0 million	Centrist	0.4872	0.6172	baseline	
30 million	Centrist	0.4853	0.5242	-0.0045	0.9035
60 million	Centrist	0.5215	0.3275	0.0288	0.4523
90 million	Centrist	0.5049	0.8213	0.0152	0.6814
120 million	Centrist	0.5013	0.9549	0.0191	0.616

**Value of Swiss trade with this country**					
0 million	Centrist	0.4246	0	baseline	
500 million	Centrist	0.5316	0.0757	0.1103	0.0003
1,000 million	Centrist	0.5455	0.0099	0.1267	0.0001
**Extreme weather event**					
Drought	Centrist	0.5213	0.3143	baseline	
Sea level rise	Centrist	0.499	0.9606	-0.0245	0.4727
Floods	Centrist	0.4799	0.3127	-0.0382	0.2673
Cyclones	Centrist	0.5039	0.8319	-0.0196	0.5217
**UN Security Council votes in line with Switzerland**					
0%	Centrist	0.4512	0.0018	baseline	
40%	Centrist	0.5087	0.5768	0.0723	0.0052
80%	Centrist	0.5439	0.0128	0.0944	0.0011
**Recipient developing country**					
Bangladesh	Right	0.5136	0.3693	baseline	
Algeria	Right	0.4561	0.0058	-0.0591	0.0256
Philippines	Right	0.5449	0.0017	0.0319	0.1701
Kenya	Right	0.485	0.3076	-0.0302	0.2014
**Number of climate migrants to accept from this country per year**					
0	Right	0.5131	0.4568	baseline	
250	Right	0.5065	0.6935	-0.0049	0.85
500	Right	0.5193	0.1809	0.0077	0.7499
750	Right	0.5446	0.0034	0.0352	0.1658
1,000	Right	0.4494	0.0014	-0.0621	0.0198
1,250	Right	0.4703	0.0981	-0.0401	0.1575
**Climate aid to give to this country (CHF) per year**					
0 million	Right	0.5055	0.7129	baseline	
30 million	Right	0.5014	0.916	-0.0004	0.9868
60 million	Right	0.4843	0.274	-0.0149	0.5282
90 million	Right	0.5063	0.6541	0.0029	0.8975
120 million	Right	0.5024	0.8689	-0.0055	0.8173
**Value of Swiss trade with this country**					
0 million	Right	0.4615	0.0001	baseline	
500 million	Right	0.5144	0.1452	0.0567	0.0009
1,000 million	Right	0.5252	0.017	0.0669	0.0002
**Extreme weather event**					
Drought	Right	0.4857	0.2394	baseline	
Sea level rise	Right	0.5035	0.7788	0.0167	0.4065
Floods	Right	0.5024	0.846	0.0175	0.3761
Cyclones	Right	0.5085	0.4846	0.0227	0.2506
**UN Security Council votes in line with Switzerland**					
0%	Right	0.4669	0.0009	baseline	
40%	Right	0.5009	0.9282	0.0333	0.0503
80%	Right	0.5334	0.0007	0.0661	0.0001
Number of observations				9848	
R2				0.0219	
Adj.R2				0.0164	

Note: Standard errors for the computation of p-values were clustered by respondent id. For MM estimates that are derived from AMCEs (for details, see Leeper et al., 2018), p-values are computed under null hypothesis that the estimate is equal to 0.5 for the binary choice outcome where respondents choose the preferred policy package when presented with two policy pairs with randomized attribute levels. The label for the attribute level 'Percentage of this country's votes in line with Switzerland's position at the UN Security Council' was replaced with to 'UN Security Council votes in line with Switzerland' for better readability.
